# ﻿Water mite diversity from southwestern Türkiye through the lens of the DNA barcodes, with the description of one new species (Acari, Hydrachnidia)

**DOI:** 10.3897/zookeys.1232.142699

**Published:** 2025-03-18

**Authors:** Vladimir Pešić, Andrzej Zawal, Pınar Gülle, İskender Gülle, Milica Jovanović, Aleksandra Bańkowska, Stanisław Musielak, Harry Smit

**Affiliations:** 1 Department of Biology, University of Montenegro, Džordža Vašingtona b.b., 81000 Podgorica, Montenegro; 2 Institute of Marine and Environmental Sciences, Center of Molecular Biology and Biotechnology, University of Szczecin, Wąska 13, 71–415 Szczecin, Poland; 3 Faculty of Science and Arts, Burdur Mehmet Akif Ersoy University, Burdur, Turkiye; 4 Institute of Biology, University of Szczecin, Wąska 13, 71-415 Szczecin, Poland; 5 Naturalis Biodiversity Center, P.O. Box 9517, 2300 RA Leiden, Netherlands; 6 Museums Victoria Research Institute, Museums Victoria, GPO Box 666, Melbourne, VIC 3001, Australia

**Keywords:** Cytochrome c oxidase subunit I (COI), new records, new species, running waters, springs, taxonomy, water mites

## Abstract

This study presents the molecular and morphological results from an analysis of water mites collected in southwestern Türkiye. 83 COI barcodes are provided, clustered into 40 BINs, with 23 BINs being unique and deposited for the first time in the Barcode of Life Data Systems (BOLD). The first DNA barcodes for eight water mite species are uploaded into the BOLD database. In total, 34 water mite species were identified and one of them, *Iranothyasmarismortui* (Gerecke, 1999) is newly reported from Türkiye. *Iranothyasalhajarica* Pešić, Gerecke & Smit, 2009 is excluded from the fauna of Türkiye. *Sperchonfundamentalis* Bader & Sepasgozarian, 1980, a species previously synonymized with *S.glandulosus* Koenike, 1886 is resurrected as a valid species. One species, *Atractidesturani* Pešić, Zawal, Gülle & Smit, **sp. nov**. (Hygrobatidae), is described as new to science.

## ﻿Introduction

The knowledge of water mites from Türkiye is still insufficient. The checklist published by Erman et al. (2010, 2019) listed 335 species in 62 genera and 25 families and summarized all previous research on water mites in Türkiye. In recent years, an increased number of regional and/or international DNA barcoding initiatives resulted in the formation and curation of water mites DNA barcode libraries (e.g., Montenegro, Pešić et al. 2021a; Norway, Gerecke et al. 2022; Corsica, Pešić and Smit 2022; Portugal, Pešić et al. 2024). The public dataset of barcodes of water mites of Iran and Türkiye (available at https://doi.org/10.5883/DS-TRIRHYD; Pešić et al. 2021b, 2022, 2023a) is still modest and includes 249 public sequences (including the sequences of this study), 188 of these are from Türkiye. As emphasized by Pešić et al. (2023d, 2024) formation of such barcode libraries and their further continuous improvement by expanding their taxonomic and geographical coverage provides is on one hand a basis for a deeper understanding of the diversity of water mites in particular areas. On the other hand, it is a valuable contribution to the integrative taxonomic research of challenging groups of species, especially for detecting cryptic or pseudocryptic species.

This paper is based on material collected in southwestern Anatolia (mainly Burdur and Isparta provinces) in 2024. As a result of this investigation, we describe one species new to science.

## ﻿Materials and methods

Water mites were collected by hand netting and immediately preserved in 96% ethanol for the purpose of molecular analyses. The specimens used for molecular study are listed in Table 1. After non-destructive DNA extraction, the specimen vouchers were stored in 96% ethanol and morphologically examined. Some of these vouchers were dissected and slide mounted in Faure’s medium, while the rest was transferred to Koenike’s fluid.

**Table 1.** Details on DNA barcoded specimens, including localities and coordinates of sampling sites, sample codes and the barcode index number codes (^N^ indicates a new BIN that contains only sequences from this study). BOLD data presented here was last accessed on 10 May 2024.

Locality	Coordinates	Sample ID	Process ID	BIN
**Hydrachnidae Leach, 1815**				
***Hydrachnaglobosa* (De Geer, 1778)**				
Burdur, Gölhisar Lake	37.1227°N, 29.599937°E	CCDB-48501-A04	HYDCG099-24	BOLD:ACI2447
		CCDB-48501-A05	HYDCG100-24	
**Hydrodromidae Viets, 1936**				
***Hydrodromatorrenticola* (Walter, 1908)**				
Antalya, pond	36.87547°N, 30.8454°E	CCDB-48498-C04	HYDCG028-24	^N^ BOLD:AGG7908
		CCDB-48498-C05	HYDCG029-24	
		CCDB-48498-C08	HYDCG032-24	BOLD:AFC2822
**Hydryphantidae Piersig, 1896**				
***Protzialongiacetabulata* Gülle & Boyaci, 2014**				
Burdur, Karacaören, stream	37.327335°N, 30.869408°E	CCDB-48498-F01	HYDCG061-24	BOLD:AEH8558
Isparta, Yazılıkanyon Tabiat Parkı, stream	37.46882°N, 30.919449°E	CCDB-48498-G10	HYDCG082-24	
		CCDB-48498-G05	HYDCG077-24	
Burdur, waterfall	37.33291°N, 30.879221°E	CCDB-48498-E01	HYDCG049-24	
***Protziavietsi* Özkan, 1982**				
Burdur, Söbüce, stream	37.287872°N, 30.067743°E	CCDB-48498-A05	HYDCG005-24	^N^ BOLD:AGG3760
Söbüce, rheocrenic spring	37.295727°N, 30.089523°E	CCDB-48498-A06	HYDCG006-24	
Burdur, Kemer, helocrenic spring	37.301468°N, 30.097061°E	CCDB-48498-B02	HYDCG014-24	
		CCDB-48498-B03	HYDCG015-24	
Isparta, Yazılıkanyon Tabiat Parkı, stream	37.46882°N, 30.919449°E	CCDB-48498-G04	HYDCG076-24	
***Trichothyaspetrophila* (Michael, 1895)**				
Burdur, waterfall	37.33291°N, 30.879221°E	CCDB-48498-D12	HYDCG048-24	^N^ BOLD:AGG3771
***Iranothyasmarismortui* (Gerecke, 1999)**				
Burdur, helocrenic spring near Burdur lkae	37.733643°N, 30.112862°E	CCDB-48498-D05	HYDCG041-24	^N^ BOLD:AGG3782
		CCDB-48498-D06	HYDCG042-24	
		CCDB-48498-D07	HYDCG043-24	
**Lebertiidae Thor, 1900**				
***Lebertiaglabra* Thor, 1897**				
Burdur, Söbüce, first order stream	37.295727°N, 30.089523°E	CCDB-48498-A10	HYDCG010-24	BOLD:ACS0595
		CCDB-48498-A11	HYDCG011-24	
Burdur, waterfall and outflow	37.33291°N, 30.879221°E	CCDB-48498-E06	HYDCG054-24	
Burdur, Akyayla, spring	37.482956°N, 30.326647°E	CCDB-48498-F12	HYDCG072-24	
Burdur, Söbüce, stream	37.287872°N, 30.067743°E	CCDB-48498-H06	HYDCG090-24	
***Lebertiarivulorum* K. Viets, 1933**				
Burdur, Sazak, spring	37.544933°N, 29.94381°E	CCDB-48498-B06	HYDCG018-24	^N^ BOLD:AGG5208
Burdur, Kestel, canal	37.429718°N, 30.399193°E	CCDB-48498-H11	HYDCG095-24	
Burdur, Çavdir, spring	37.14478°N, 29.656534°E	CCDB-48498-E12	HYDCG060-24	
**Sperchontidae Thor, 1900**				
***Sperchonbeneckei* Bader & Sepasgosarian, 1982**				
Isparta, Yazılıkanyon Tabiat Parkı, stream	37.46882°N, 30.919449°E	CCDB-48498-G01	HYDCG073-24	BOLD:AED2730
***Sperchoncompactilis* Koenike, 1911**				
Burdur, Söbüce, stream	37.287872°N, 30.067743°E	CCDB-48498-H05	HYDCG089-24	BOLD:ACS1036
Burdur, canal	37.429718°N, 30.399193°E	CCDB-48498-H10	HYDCG094-24	
***Sperchonthienemanni* Koenike, 1907**				
Burdur, Söbüce, stream	37.287872°N, 30.067743°E	CCDB-48498-A07	HYDCG007-24	BOLD:AES4247
		CCDB-48498-A03	HYDCG003-24	
Burdur, Akyayla, spring	37.482956 °N, 30.326647 °E	CCDB-48498-F09	HYDCG069-24	^N^ BOLD:AGG3777
***Sperchonpapillosus* Thor, 1901**				
Isparta, Çukurköy, stream	37.651257°N, 30.81791°E	CCDB-48498-D03	HYDCG039-24	^N^ BOLD:AGH7685
Isparta, Kışlaköy	37.66509°N, 30.725111°E	CCDB-48501-A12	HYDCG107-24	
***Sperchonserapae* Boyaci, Gülle & Özkan, 2012**				
Burdur, Akyayla, spring	37.482956°N, 30.326647°E	CCDB-48498-F10	HYDCG070-24	^N^ BOLD:AGG3776
***Sperchonsetiger* Thor, 1898**				
Burdur, Çavdir, spring	37.14478°N, 29.656534°E	CCDB-48498-E11	HYDCG059-24	^N^ BOLD:AGH7686
Burdur, Kestel, canal	37.429718°N, 30.399193°E	CCDB-48498-H09	HYDCG093-24	^N^ BOLD:AGG3936
**Family Torrenticolidae Piersig, 1902**				
***Torrenticolabaueri* Bader & Sepasgozarian, 1987**				
Burdur, Karacaören, stream	37.327335°N, 30.869408°E	CCDB-48498-F04	HYDCG064-24	BOLD:AFG4655
		CCDB-48498-F06	HYDCG066-24	
***Monatractidesstadleri* (Walter, 1924)**				
Burdur, waterfall and outflow	37.33291°N, 30.879221°E	CCDB-48498-E03	HYDCG051-24	BOLD:AGC6044
**Limnesiidae Thor, 1900**				
***Limnesiafulgida* Koch, 1836**				
Antalya, limnocrene spring	37.09568°N, 30.58095°E	CCDB-48498-E07	HYDCG055-24	^N^ BOLD:AGG4400
		CCDB-48498-E09	HYDCG057-24	
**Hygrobatidae Koch, 1842**				
***Atractidesfonticola* K. Viets, 1920**				
Isparta, Kışlaköy	37.66509°N, 30.725111°E	CCDB-48501-A08	HYDCG103-24	^N^ BOLD:AGG3788
Burdur, Akyayla, spring	37.515774°N, 30.35459°E	CCDB-48498-B11	HYDCG023-24	
***Atractidesgraecus* K. Viets, 1950**				
Burdur, Karacaören, stream	37.327335°N, 30.869408°E	CCDB-48498-F07	HYDCG067-24	^N^ BOLD:AGG3781
***Atractidesinflatipalpis* K. Viets, 1950**				
Burdur, Akyayla, spring	37.515774°N, 30.35459°E	CCDB-48498-B12	HYDCG024-24	^N^ BOLD:AGG3787
		CCDB-48498-C02	HYDCG026-24	
***Atractideslunipes* Lundblad, 1956**				
Burdur, Karacaören, stream	37.327335°N, 30.869408°E	CCDB-48498-F08	HYDCG068-24	^N^ BOLD:AGG3780
***Atractidesnikooae* Pesic, 2004**				
Burdur, Karamusa stream	37.186405°N, 29.75374°E	CCDB-48498-D10	HYDCG046-24	^N^ BOLD:AGG3766
		CCDB-48498-D11	HYDCG047-24	
Burdur, Çavdir, spring	37.14478°N, 29.656534°E	CCDB-48498-E10	HYDCG058-24	
***Atractidesrobustus* (Sokolow, 1940)**				
Isparta, Yazılıkanyon Tabiat Parkı, stream	37.46882°N, 30.919449°E	CCDB-48498-G02	HYDCG074-24	BOLD:AEK3669
Isparta, Çukurköy, stream	37.651257°N, 30.81791°E	CCDB-48498-D04	HYDCG040-24	^N^ BOLD:AGH5609
***Atractidessubasper* Koenike, 1902**				
Burdur, Söbüce, stream	37.287872°N, 30.067743°E	CCDB-48498-H02	HYDCG086-24	^N^ BOLD:AGG3778
***Atractidesturani* sp. nov.**				
Burdur, Söbüce, stream	37.287872°N, 30.067743°E	CCDB-48498-A09	HYDCG009-24	^N^ BOLD:AGG3768
Burdur, Akyayla, spring	37.482956°N, 30.326647°E	CCDB-48498-F11	HYDCG071-24	^N^ BOLD:AGG3774
***Hygrobateslongipalpis* (Hermann, 1804)**				
Burdur, Sazak, spring	37.544933°N, 29.94381°E	CCDB-48498-B05	HYDCG017-24	BOLD:AES0232
		CCDB-48498-B07	HYDCG019-24	
Burdur, Dereköy, spring	37.42846°N, 29.809637°E	CCDB-48498-B10	HYDCG022-24	
Antalya, limnocrene spring	37.09568°N, 30.58095°E	CCDB-48498-E08	HYDCG056-24	
Burdur, Kayali, limnocrene	37.306606°N, 29.931082°E	CCDB-48501-A09	HYDCG104-24	
Isparta, Kışlaköy	37.66509°N, 30.725111°E	CCDB-48501-A11	HYDCG106-24	
***Hygrobatespersicus* Pešić & Asadi, 2017**				
Antalya, Düden river	36.959763°N, 30.731194°E	CCDB-48498-C09	HYDCG033-24	BOLD:ACB5533
		CCDB-48498-C10	HYDCG034-24	
		CCDB-48498-C11	HYDCG035-24	
		CCDB-48498-C12	HYDCG036-24	
Isparta, Kışlaköy	37.66509°N, 30.725111°E	CCDB-48501-A06	HYDCG101-24	
		CCDB-48501-A07	HYDCG102-24	
***Hygrobatesquanaticola* Schwoerbel & Sepasgozarian, 1976**				
Burdur, Kuzköy, spring	37.55402°N, 30.440313°E	CCDB-48498-A02	HYDCG002-24	BOLD:AEM9575
		CCDB-48501-A10	HYDCG105-24	
		CCDB-48498-A01	HYDCG001-24	^N^ BOLD:AGG3789
Burdur, Dereköy, spring	37.42846°N, 29.809637°E	CCDB-48498-B09	HYDCG021-24	
Burdur, canal	37.429718°N, 30.399193°E	CCDB-48498-H08	HYDCG092-24	
**Unionicolidae Oudemans, 1909**				
***Neumaniaimitata* Koenike, 1908**				
Antalya, pond	36.87547°N, 30.8454°E	CCDB-48498-C06	HYDCG030-24	^N^ BOLD:AGG4333
		CCDB-48498-C07	HYDCG031-24	
***Neumanialimosa* (Koch, 1836)**				
Burdur, Duger, limnocrene spring	37.574345°N, 30.021276°E	CCDB-48501-C04	HYDCG123-24	BOLD:AEF5902
**Pionidae Thor, 1900**				
***Pionaalpicola* (Neuman, 1880)**				
Uylupinar, limnocrene spring	37.10993°N, 29.613293°E	CCDB-48501-A03	HYDCG098-24	BOLD:ACR9570
**Arrenuridae Thor, 1900**				
***Arrenuruscompactus* Piersig, 1894**				
Uylupinar, limnocrene spring	37.10993 °N, 29.613293 °E	CCDB-48501-A01	HYDCG096-24	BOLD:AEJ6492
***Arrenurusfontinalis* K. Viets, 1920**				
Burdur, Kemer, helocrenic spring	37.301468°N, 30.097061°E	CCDB-48498-A12	HYDCG012-24	^N^ BOLD:AGH5781
		CCDB-48498-B01	HYDCG013-24	
		CCDB-48498-B04	HYDCG016-24	
Burdur, Akyayla, spring	37.515774°N, 30.35459°E	CCDB-48498-C03	HYDCG027-24	
***Arrenurussuecicus* Lundblad, 1917**				
Burdur, limnocrene spring	37.10993°N, 29.613293°E	CCDB-48501-A02	HYDCG097-24	BOLD:AAV9863

Morphological nomenclature follows Gerecke et al. (2016). The holotype of the new species is deposited in Naturalis Biodiversity Center in Leiden (**RMNH**). In the section ′Material examined’, collecting site abbreviations are derived from the geographical database of the first and second authors.

All measurements are given in μm. The photographs of selected structures were made using a camera on Samsung Galaxy smartphone. The following abbreviations are used:

**Ac-1-3** first to third acetabula;

**Cx-I-IV** first to fourth coxae;

**Dgl-4** dorsoglandularia 4;

**dL** dorsal length;

**H** height;

**I-L-4-6** fourth-sixth segments of first leg;

**L** length;

**lL** lateral length;

**mL** medial length;

**P-1-P-5** palp segment 1-5;

**S-1** proximal large ventral seta at I-L-5;

**S-2** distal large ventral seta at I-L-5;

**Vgl-1** ventroglandulare 1;

**Vgl-2** ventroglandulare 2;

**vL** ventral length;

**W** width.

### ﻿Molecular and DNA barcode analyses

Molecular analyses were conducted at the Canadian Centre for DNA Barcoding, Guelph, Canada (CCDB; http://ccdb.ca/). In the later institution, the specimens were sequenced for the barcode region of COI using standard invertebrate DNA extraction, amplification, and sequencing protocols (see for details: Ivanova et al. 2007; Ivanova and Grainger 2007a, b).

Consensus sequences were made available in the Barcode of Life Data Systems (BOLD) (Ratnasingham and Hebert 2007). The Barcode Index Numbers (BIN), grouping DNA sequences based on the Refined Single Linkage (RESL) analysis performed in BOLD (Ratnasingham and Hebert 2013), were obtained. Relevant voucher information, photos, and newly generated DNA barcodes are publicly accessible through https://doi.org/10.5883/DS-TRIRHYD in BOLD. Data related to each BIN, which are often considered proxies for species, including the minimum *p*-distance to the nearest neighboring BIN, was estimated using BOLD tools. In this study DNA was extracted from 83 specimens from Türkiye listed in Table 1. For all other species, COI sequence data were downloaded from the respective sequence data archives.

Sequence alignments were performed using MUSCLE (Edgar 2004). Intra- and interspecific genetic distances were calculated based on the Kimura 2-parameter model (K2P; Kimura 1980), using MEGA 11 software (Tamura et al. 2021). The latter software was used to calculate Neighbor-Joining (NJ) trees based on K2P distances (standard for barcoding studies) using pairwise deletion for missing data. Branch support was calculated using nonparametric bootstrap (Felsenstein 1985) with 1000 replicates and shown next to the branches.

Additionally, the sequence data were analyzed using the Assemble Species by Automatic Partitioning (ASAP) method (Puillandre et al. 2012). We used the online ASAP version (https://bioinfo.mnhn.fr/abi/public/asap/asapweb.html) with default settings and K2P distance model.

## ﻿Results and discussion

We generated 83 DNA barcodes from 34 water mite species collected in southwestern Türkiye during our collecting trip in April 2024. The collected water mites represent 11 families and 15 genera. The most sequence-rich family was Hygrobatidae with 31 sequences (14 BINs), followed by Hydryphantidae with 13 sequences (4 BINs), and Sperchontidae with 11 sequences (8 BINs). Some families were rare, such as Hydrachnidae, Hydrodromidae, and Pionidae, represented by a single BIN each.

The resulting sequences clustered into 40 BINs, with 23 BINs (57.5%) being unique and deposited for the first time in BOLD. Two BINs were detected for six species, i.e., *Hydrodromatorrenticola* (Walter, 1908) (BOLD:AFC2822, BOLD:AGG7908), *Sperchonthienemanni* Koenike, 1907 (BOLD:AES4247, BOLD:AGG3777), *S.setiger* Thor, 1898 (BOLD:AGG3936, BOLD:AGH7686), *Atractidesrobustus* (Sokolow, 1940) (BOLD:AGH5609, BOLD:AEK3669), *A.turani* sp. nov. (BOLD:AGG3768, BOLD:AGG3768) and *Hygrobatesquanaticola* Schwoerbel & Sepasgozarian, 1976 (BOLD:AGG3789, BOLD:AEM9575). Our study provided the first DNA barcodes for *Protziavietsi* Özkan, 1982 (BOLD:AGG3760), *Iranothyasmarismortui* (Gerecke, 1999) (BOLD:AGG3782), *Trichothyaspetrophila* (Michael, 1895) (BOLD:AGG3771), *Sperchonserapae* Boyaci, Gülle & Özkan, 2012 (BOLD:AGG3776), *Atractidesgraecus* K. Viets, 1950 (BOLD:AGG3781), *A.inflatipalpis* K. Viets, 1950 (BOLD:AGG3787), *A.lunipes* Lundblad, 1956 (BOLD:AGG3780), and *A.nikooae* Pesic, 2004 (BOLD:AGG3766).

Our findings added the first record of *Iranothyasmarismortui* (Gerecke, 1999) for Türkiye. One species of the genus *Atractides* (*A.turani* sp. nov.) is described as new to science.

In summary, even though sampling was conducted in a short period (22–27 April 2024), this study exemplifies the high molecular diversity of water mites of southwestern Türkiye and at the same time highlights the need to intensify further studies with the aim of the generation and curation of DNA barcode reference libraries at the regional level.

### ﻿Systematics

#### ﻿Family Hydrachnidae Leach, 1815

**Genus *Hydrachna* Müller, 1776**

##### ﻿ Hydrachna globosa

Taxon classificationAnimaliaTrombidiformesHydrachnidae

(De Geer, 1778)

542F61DB-D475-52ED-8868-8DB6841423DA

###### Material examined.

**Burdur** • TR21-2024, Gölhisar Lake, 37.1227°N, 29.599937°E, 26 Apr. 2024, leg. Pešić, Zawal, Saboori, Gülle & Gülle, 2♀ (sequenced).

###### Remarks.

The sequences obtained from the specimens from Türkiye fall into BOLD:ACI2447, which, in addition to the specimens used in this study for molecular analysis, includes specimens of *H.globosa* from the Netherlands and Norway, available in the BOLD database.

###### Distribution.

Palaearctic.

#### ﻿Family Hydrodromidae Viets, 1936

**Genus *Hydrodroma* Koch, 1837**

##### ﻿ Hydrodroma torrenticola

Taxon classificationAnimaliaTrombidiformesHydrodromidae

(Walter, 1908)

3AE025F8-6F36-56FA-B2BF-3165CA947139

###### Material examined.

**Antalya** • TR29-2024 Aksu, pond near Antalya city, 36.87547°N, 30.8454°E, 27 Apr. 2024 leg. Pešić, Zawal, Gülle & Gülle, 3♀ (sequenced).

###### Remarks.

The sequences obtained from the three females collected in a pond near Antalya cluster within two BINs. One specimen falls within BOLD:AFC2822, which in addition to the specimen from this study, includes one specimen of *H.torrenticola* from Norway, with the nearest neighboring BIN being BOLD:ACI2515, which includes specimens of *H.torrenticola* from Montenegro from which it differs by 3.24% *p*-distance. Two specimens of *H.torrenticola* from the same locality, form the unique BOLD:AGG7908, with the nearest neighboring BIN being BOLD:AFC2822, from which it differs by 3.86% *p*-distance.

###### Distribution.

Central, Western, and Southern Europe, Türkiye.

#### ﻿Family Hydryphantidae Piersig, 1896

**Genus *Protzia* Piersig, 1896**

##### ﻿ Protzia longiacetabulata

Taxon classificationAnimaliaTrombidiformesHydryphantidae

Gülle & Boyaci, 2014

7C443170-CE9F-52D4-8C67-712F2F096648

###### Material examined.

**Isparta** • TR18-2024, Yazılıkanyon Tabiat Parkı, stream from cave (moss), 37.46882°N, 30.919449°E, 25 Apr. 2024, leg. Pešić, Zawal, Gülle & Gülle, 1♂, 1♀ (sequenced). **Burdur** • TR19-2024 waterfall and outflow, 37.33291°N, 30.879221°E, 25 Apr. 2024, leg. Pešić, Zawal, Gülle & Gülle, 1♀ (sequenced) • TR20-2024, Karacaören, stream, 37.327335°N, 30.869408°E, 25 Apr. 2024, Pešić, Zawal, Gülle & Gülle, 1♀ (sequenced).

###### Remarks.

The sequenced specimens from this study cluster together with specimens, collected from the Sütçüler stream in Isparta Province, morphologically assigned by Pešić et al. (2021c) to *P.longiacetabulata*. The latter rhitrobiontic species was originally described by Gülle and Boyaci (2014) from the Dim stream in Alanya province. The examined specimens of *P.longiacetabulata* belongs to BIN (BOLD:AEH8558), with a *p*-distance of 3.85% to the nearest BIN being BOLD:AEH8557 which groups two sequences of the latter species from Isparta Province.

###### Distribution.

Türkiye.

##### ﻿ Protzia vietsi

Taxon classificationAnimaliaTrombidiformesHydryphantidae

Özkan, 1982

E6F79AD0-B612-5361-8BFD-5409F26E42AE

###### Material examined.

**Burdur** • TR9 Kemer, helocrenic spring 37.301468°N, 30.097061°E, 22 Apr. 2024, leg. Pešić, Zawal, Gülle & Gülle, 2♀ (sequenced) • TR10-2024 Söbüce, rheocrenic spring, 37.295727°N, 30.089523°E, 24 Apr. 2024, leg. Pešić, Zawal, Gülle & Gülle, 1♀ (sequenced) • TR11-2024, Söbüce, first order stream, 37.287872°N, 30.067743°E, 24 Apr. 2024, leg. Pešić, Zawal, Gülle & Gülle, 1♀ (sequenced). **Isparta** • TR18-2024, Yazılıkanyon Tabiat Parkı, stream from cave (mosses), 37.46882°N, 30.919449°E, 25 Apr. 2024, leg. Pešić, Zawal, Gülle & Gülle, 1♀ (sequenced).

###### Remarks.

The examined specimens from southwestern Türkiye used in this study for molecular analysis match the description of *P.vietsi.* The latter species, originally described by Oezkan (1982) from Erzurum province belongs to the *eximia* species group (see Gerecke 1996 for discussion on diagnostic features of *P.vietsi*). The molecularly studied specimens from Burdur and Isparta forms a unique BIN (BOLD:AGG3760).

###### Distribution.

Türkiye.

#### ﻿Genus *Iranothyas* Bader, 1984

##### ﻿ Iranothyas marismortui

Taxon classificationAnimaliaActinedidaHydryphantidae

(Gerecke, 1999)

D910ABD6-D871-5F3C-91E7-9334B501D52E

###### Material examined.

**Burdur** • TR4-2024 helocrenic spring near Burdur Lake, 37.733643°N, 30.112862°E, 23 Apr. 2024, leg. Pešić, Zawal, Gülle & Gülle 3♀ (sequenced).

###### Remarks.

The genus *Iranothyas* includes three species, *Iranothyascircularis* (Schwoerbel & Sepasgozarian, 1976), known from a single female from the type locality in western Iran (Schwoerbel and Sepasgozarian 1976), *Iranothyasalhajarica* Pešić, Gerecke & Smit, 2009, a species originally described from Oman (Pešić et al. 2009), and *I.marismortui* (Gerecke, 1999), a species so far known only from Israel (Gerecke 1999).

In regard to the shape of the frontal shield (in agreement with *Iranothyascircularis*, nearly circular, outline equally rounded, without a posteromedial extension as in *I.alhajarica*) and position of Ac-2 (in agreement with *I.alhajarica*, halfway between Ac-1 and Ac-3, not close to Ac-3 as in *I.circularis*) the examined specimens from a helocrenic spring located on the west coast of the Burdur Lake matches the description of *I.marismortui* (Gerecke, 1999). Genetic data indicate that these specimens form a unique BIN (BOLD:AGG3782).

Gülle et al. (2017) reported the presence of *Iranothyasalhajarica* Pešić, Gerecke & Smit, 2009 from the same spring located on the west coast of the Burdur Lake (Fig. 6C) where we found an *Iranothyas* population of here assigned to *I.marismortui*. Therefore, we assume that the record of *I.alhajarica* in Gülle et al. (2017) refers to *I.marismortui*, indicating that *I.alhajarica* should be excluded for Turkish fauna. Gülle et al. (2017) mentioned that the larvae of the *Iranothyas* population on the aforementioned locality were found as parasites on the hydrophilic beetle *Laccobiusgracilis* Motschulsky, 1855.

###### Distribution.

Israel, Türkiye.

#### ﻿Genus *Trichothyas* K. Viets, 1926

##### ﻿ Trichothyas (Lundbladia) petrophila

Taxon classificationAnimaliaTrombidiformesHydryphantidae

(Michael, 1895)

06B6F790-A5CF-520D-BBE4-47D6146A489C

###### Material examined.

**Burdur** • TR19-2024 waterfall and outflow, 37.33291°N, 30.879221°E, 25 Apr. 2024, leg. Pešić, Zawal, Gülle & Gülle, 1♀ (sequenced).

###### Remarks.

The single specimen from southwestern Türkiye, molecularly analyzed in this study, matches the description of *T.petrophila.* This specimen forms a unique BIN (BOLD:AGG3771).

###### Distribution.

Southern and Western Europe, Great Britain, Balkan, Türkiye.

#### ﻿Family Lebertiidae Thor, 1900

**Genus *Lebertia* Neuman, 1880**

##### ﻿ Lebertia (Lebertia) glabra

Taxon classificationAnimaliaTrombidiformesLebertiidae

Thor, 1897

74D230F7-C315-550A-B5FE-CF5B0BA9DC0E

###### Material examined.

**Burdur** • TR2-2024 Akyayla, rheocrenic spring, 37.482956°N, 30.326647°E, 22 Apr. 2024, leg. Pešić, Zawal, Gülle & Gülle, 1♂ (sequenced); **Burdur** • TR10-2024 Söbüce, first order stream, 37.295727°N, 30.089523°E, 24 Apr. 2024, leg. Pešić, Zawal, Gülle & Gülle, 2♀ (sequenced) • TR11-2024, Söbüce, stream, 37.287872°N, 30.067743°E, 24 Apr. 2024, leg. Pešić, Zawal, Gülle & Gülle, 1♂ (sequenced) • TR19-2024 waterfall and outflow, 37.33291°N, 30.879221°E, 25 Apr. 2024 leg. Pešić, Zawal, Gülle & Gülle, 1♀ (sequenced).

###### Remarks.

The specimens from Burdur match the description of *L.glabra*, a species widely distributed in the Western Palaearctic (Di Sabatino et al. 2010). The Turkish specimens were clustered in BOLD:ACS0595, which includes specimens of *L.glabra* from the Netherlands, Bulgaria, Norh Macedonia, Montenegro, Italy, Poland, Slovakia, Romania, Germany, Austria, Serbia, Norway, Bosnia and Herzegovina, and Türkiye.

###### Distribution.

Western Palaearctic.

##### ﻿ Lebertia (Lebertia) rivulorum

Taxon classificationAnimaliaTrombidiformesLebertiidae

K. Viets, 1933

2D4A0E6C-FAF3-51E4-8274-FF2D64078EE6

###### Material examined.

**Burdur** • TR6-2024 Sazak, rheocrenic spring, 37.544933°N, 29.94381°E, 23 Apr. 2024, leg. Pešić, Zawal, Gülle & Gülle, 1♀ (sequenced) • TR23-2024, Çavdir, spring, 37.14478°N, 29.656534°E, 26 Apr. 2024, leg. Pešić, Zawal, Gülle & Gülle, 1♀ (sequenced) • TR26-2024 Kestel, canal with fast-flowing water, 37.429718°N, 30.399193°E, 27 Apr. 2024, leg. Pešić, Zawal, Gülle & Gülle, 1♀ (sequenced).

###### Remarks.

The examined specimens from Burdur, molecularly analyzed in this study, matches the description of *L.rivulorum.* These specimens form a unique BIN (BOLD:AGG5208). The *p*-distance to its nearest neighboring BIN (BOLD:AED9196), which includes specimens of *L.rivulorum* from North Macedonia, was estimated at 6.31%.

###### Distribution.

Central, Western, and Southern Europe, Türkiye.

#### ﻿Family Sperchontidae Thor, 1900

**Genus *Sperchon* Kramer, 1877**

##### ﻿ Sperchon beneckei

Taxon classificationAnimaliaTrombidiformesSperchontidae

Bader & Sepasgosarian, 1982

CB86FCAB-D23E-5A07-BBC8-1CBCD872E8C7

###### Material examined.

**Isparta** • TR18-2024, Yazılıkanyon Tabiat Parkı, stream from cave (moss), 37.46882°N, 30.919449°E, 25 Apr. 2024, leg. Pešić, Zawal, Gülle & Gülle, 1♀ (sequenced).

###### Remarks.

The sequence obtained from one specimen from Isparta fell into BINBOLD:AED2730, which besides the specimen from this study, includes one specimen from Iran morphologically assigned to *S.beneckei.* The latter species was originally described from Iran. Asadi et al. (2010) synonymized the species with *S.algeriensis* Lundblad, 1942, a species described from northern Africa (Lundblad 1942) and subsequently recorded from many sites in the central and western Mediterranean area (Di Sabatino et al. 2010). Recently, Pešić et al. (2024) emphasized that high genetic distance of 15.4% between Iberian populations of *S.algeriensis* and the specimen from Iran, suggests that *S.beneckei* is a distinct species and its synonymization with *S.algeriensis* should be rejected. The new findings of *S.beneckei* from southeastern Türkiye show that this species is widespread, and therefore, the known populations of *S.algeriensis* from Türkiye (e.g., Esen et al. 2013b) should be checked using molecular methods to see if they can be assigned to *S.beneckei*.

###### Distribution.

Iran, Türkiye.

##### ﻿ Sperchon (Hispidosperchon) compactilis

Taxon classificationAnimaliaTrombidiformesSperchontidae

Koenike, 1911

32C7FCC4-8E15-5F6B-9FDA-2F4797948231

###### Material examined.

**Burdur** • TR11-2024, Söbüce, first order stream, 37.287872°N, 30.067743°E, 24 Apr. 2024 leg. leg. Pešić, Zawal, Gülle & Gülle, 1♂ (sequenced) • TR26-2024 Kestel, canal with fast-flowing water, 37.429718°N, 30.399193°E, 27 Apr. 2024, leg. Pešić, Zawal, Gülle & Gülle, 1♀ (sequenced).

###### Remarks.

The sequences obtained from specimens collected in Burdur, keyed out to *S.compactilis* following Di Sabatino et al. (2010), clustered in BOLD:ACS1036, which, in addition to specimens used in this study, includes specimens of *S.compactilis* from the Netherlands, Germany, and Türkiye.

###### Distribution.

Central and SW Europe, Türkiye, Iran.

##### ﻿ Sperchon (Sperchon) thienemanni

Taxon classificationAnimaliaTrombidiformesSperchontidae

Koenike, 1907

AE9D4ABB-E20C-59DE-B5F5-80648C385D46

Fig. 1

###### Material examined.

**Burdur** • TR2-2024 Akyayla, rheocrenic spring, 37.482956°N, 30.326647°E, 22 Apr. 2024, leg. Pešić, Zawal, Gülle & Gülle, 1♀ (sequenced) • TR10-2024, Söbüce, first order stream, 37.287872°N, 30.067743°E, 24 Apr. 2024 leg. leg. Pešić, Zawal, Gülle & Gülle, 2♂ (sequenced).

###### Compared material.

*Sperchonthienemanni*: The Netherlands • Overijssel, De Lutte, 52.329°N, 6.987°E, 19 May 2012 leg. Smit 2♂, 1♀, 1♂ (NLACA054-15/RMNH.ACA.1072), 1♀ (NLACA055-15/RMNH.ACA.1073) sequenced, dissected, and slide mounted (RMNH) • Limburg, Schin op Geul: Genhoes, 50.854°N, 5.858 °E, 27 Apr. 2012 leg. Smit 3♀ (sequenced; NLACA426-15/RMNH.ACA.851, NLACA427-15/RMNH.ACA.852, NLACA428-15/RMNH.ACA.853), dissected and slide mounted (RMNH) • Limburg, Epen: Terziet, 50.755°N, 5.904°E, 27 Apr. 2012 leg. Smit 1♂ (NLACA422-15/RMNH.ACA.847), 1♀ (NLACA421-15/ RMNH.ACA.846), sequenced, dissected and slide mounted (RMNH).

###### Remarks.

The specimens sequenced from this study were clustered within two BINs. The first one, BOLD:AES4247, in addition to two specimens from this study, includes four specimens from Austria. The second BIN, BOLD:AGG3777, is unique and includes one specimen collected in a rheocrenic spring in this study. The *p*-distance between these two BINs was estimated at 2.73%. In the phylogenetic tree, the two above-mentioned BINs of *S.thienemanni* from Türkiye forms a highly supported clade which is placed (albeit with a low support) as sister to clade grouping specimens of *S.thienemanni* from the Netherlands. The latter specimens belong to BOLD:ACS0087.

**Figure 1.**
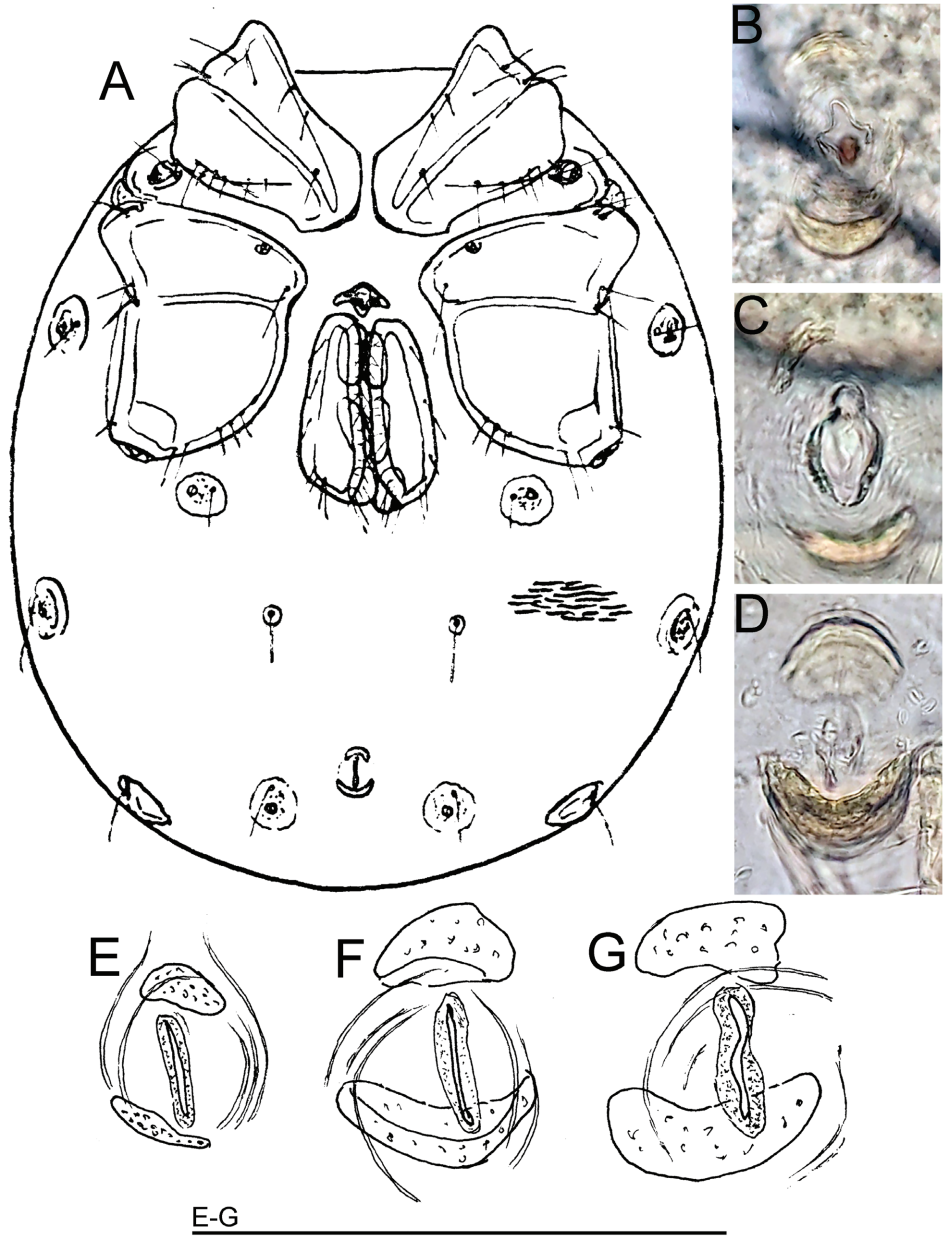
*Sperchonthienemanni*: **A** idiosoma, ventral view (from K. Viets 1936: fig. 146b) **B–G** excretory pore **B**RMNH.ACA.851, Netherlands **C**RMNH.ACA.852, Netherlands **D**RMNH.ACA.1073, Netherlands **E** ♂, CCDB-48498-A03, Türkiye **F** ♂, CCDB-48498-A07, Türkiye **G** ♀, CCDB-48498-F09, Türkiye. Scale bar: 100 μm.

In all barcoded specimens from southwestern Türkiye as well in examined specimens of *S.thienemanni* from the Netherlands belonging to BOLD:ACS0087 and BOLD:ACR9585, respectively, the excretory pore sclerotized ring was not complete, and was reduced to a separate sclerotized platelets located anterior and posterior of excretory pore, respectively (as illustrated in Fig. 1B–G). The excretory pore, not completely surrounded by a sclerite ring is well visible in K. Viets’ figure (1936: fig. 146b), but in recent water mite literature it has not been recognized as an important diagnostic character of the latter species. For example, in a key to Central European water mites (Di Sabatino et al. 2010), the excretory pore of *S.thienemanni* is described as unsclerotized without mentioning the presence of separate sclerites located anteriorly and posteriorly to the excretory pore which may lead to confusion in the identification of this species

*Sperchonthienemanni* was considered to be synonymous with *S.glandulosus* Koenike, 1886 for a long time and was only distinguished as a separate species by Szalay (1941, 1956). Following Bader (1974), and later accepted by Gerecke (1991) and Di Sabatino et al. (2010), the excretory pore in *S.glandulosus* is completely surrounded by a sclerotized ring.

**Figure 2.**
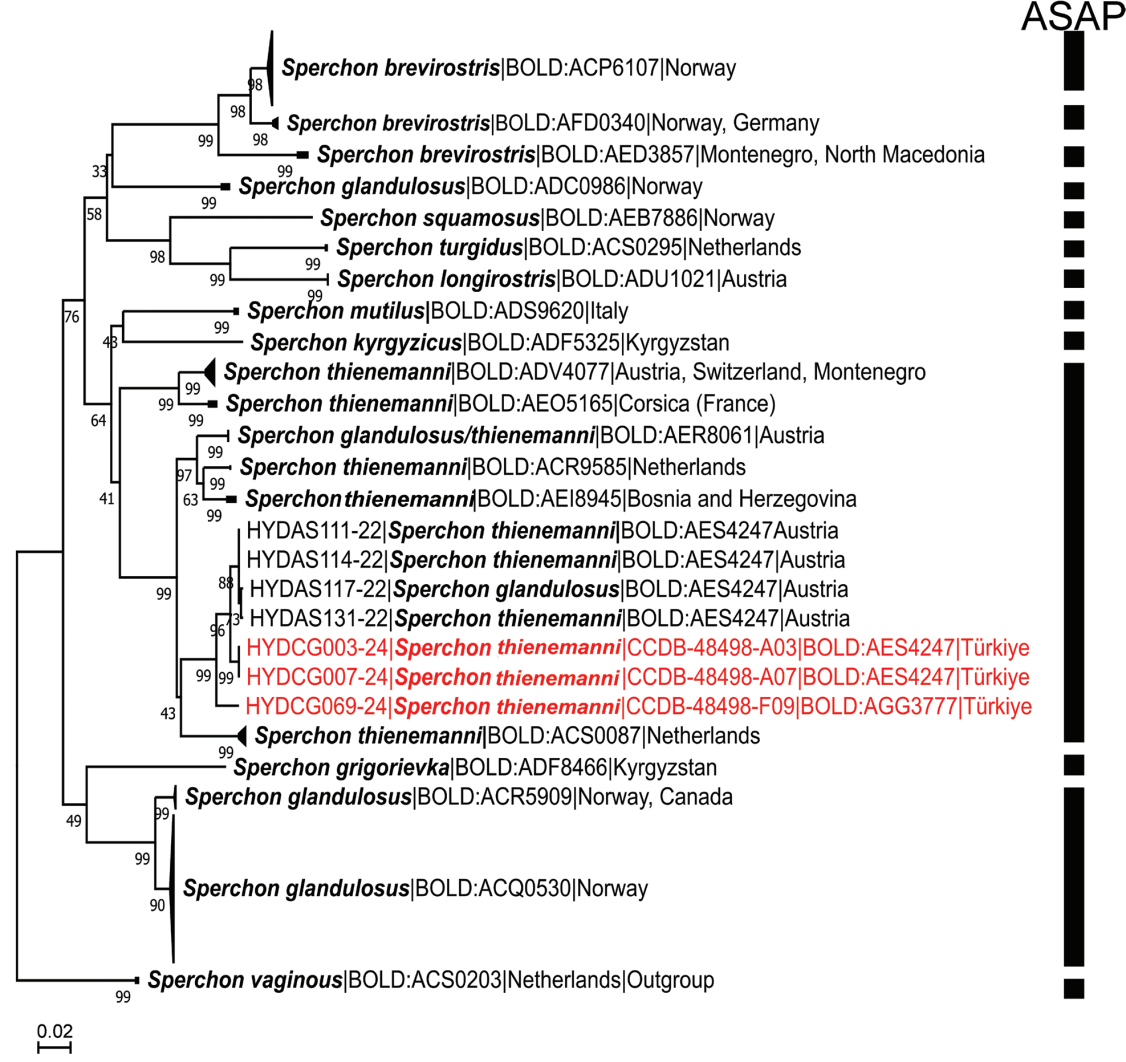
Neighbor-Joining tree of the subgenus Sperchon s. str. obtained from 161 nucleotide COI sequences listed in Suppl. material 1. *Sperchonvaginosus* Thor, 1902 from the subgenus Hispidosperchon was used to root the tree. The results of species delimitation by ASAP procedure are indicated by vertical bars. BINs are based on the barcode analysis from 4 November 2024. New sequences from this study are marked in red.

The applied ASAP procedure (see Fig. 2) grouped together the COI sequences of *S.thienemanni*-like mites belonging to the following BINs: BOLD:ADV4077 (specimens from Austria, Switzerland, and Poland available in BOLD database), BOLD:AEO5165 (specimens from Corsica), BOLD:AER8061 (specimens from Austria), BOLD:ACR9585 (specimens from Netherlands), BOLD:AEI8945 (specimens from Bosnia and Herzegovina), BOLD:ACS0087 (specimens from Netherlands), BOLD:AGG3777 (specimen from Türkiye), and BOLD:AES4247 (specimens from Türkiye and Austria).

The two lineages of *S.glandulosus*-like mites from Europe were identified as separate MOTUs (hypothetical species). The first MOTU includes Norwegian specimens of two BINs, BOLD:ACQ0530 (shared with Romania and Belgium) and BOLD:ACR5909 (shared with Canada), indicating a rather wide, and possible a circumpolar, distribution of this species. The second MOTU represented by BOLD:ADC0986 includes two specimens from Norway, with a *p*-distance of 4.99% to the closest BIN being BOLD:AEZ0976, which includes one non-identified specimen from Canada. In the phylogenetic tree, the latter BIN is placed as a sister (albeit with a low support) of clades grouping sequences of *S.brevirostris* Koenike, 1895, indicating that likely this species is phylogenetically closer to the *S.brevirostris* complex than to the *S.glandulosus* complex.

Recently, Gerecke et al. (2022) showed that DNA barcodes attributed to Norwegian *S.glandulosus* grouped into two distinct lineages, suggesting that further revision of Norwegian *glandulosus*-like mites will result in a revival of the junior synonym *S.multiplicatus* Thor, 1902, a species described from northern and eastern Norway. However, for a more sound taxonomic revision of *S.glandulosus*-like mites it is necessary to analyze molecularly more samples from a wider geographical area, preferably by including an additional genetic marker.

*Sperchonfundamentalis* Bader & Sepasgozarian, 1980, a species originally described from Iran (Bader and Sepasgozarian 1980), but later proposed to be a synonym of *S.glandulosus* by Esen et al. (2010), differs in the presence of muscle attachment plates on the dorsal and ventral sides of idiosoma (see Bader and Sepasgozarian 1980 for details). Therefore, synonymization of the latter species with *S.glandulosus* needs to be rejected and *S.fundamentalis* should be resurrected as a valid species.

###### Distribution.

Europe (except Scandinavia), Türkiye.

##### ﻿ Sperchon (Hispidosperchon) papillosus

Taxon classificationAnimaliaTrombidiformesSperchontidae

Thor, 1901

032814B2-FBCB-524B-AEE6-7282EC5F5750

###### Material examined.

**Isparta** • TR15a-2024 Kışlaköy, spring, 37.66509°N, 30.725111°E, 25 Apr. 2024 leg. leg. Pešić, Zawal, Gülle & Gülle, 1♀ (sequenced) • TR17-2024 Çukurköy, stream, 37.651257°N, 30.81791°E, 25 Apr. 2024, leg. 1♀ (sequenced).

###### Remarks.

The sequences obtained from specimens collected in Burdur, keyed to *S.papillosus* following Di Sabatino et al. (2010), forms a unique BOLD:AGH7685. The *p*-distance to its nearest neighboring BIN (BOLD:AED2135), which includes specimens from Iran morphologically assigned to *S.papillosus*, was estimated at only 1.53%.

###### Distribution.

Europe, Türkiye, Iran.

##### ﻿ Sperchon (Hispidosperchon) serapae

Taxon classificationAnimaliaTrombidiformesSperchontidae

Boyaci, Gülle & Özkan, 2012

5DAB7E70-0DB0-5676-B8A2-583FE447BBBE

###### Material examined.

**Burdur** • TR2-2024 Akyayla, rheocrenic spring, 37.482956°N, 30.326647°E, 22 Apr. 2024, leg. Pešić, Zawal, Gülle & Gülle, 1♀ (sequenced), one palp dissected and slide mounted.

###### Remarks.

The single examined female in this study matches the description of *Sperchonserapae*, a species originally described by Boyaci et al. (2012) from Taurus Mountains in southern Turkey. This specimen forms a unique BIN (BOLD:AGG3776). The *p*-distance to its nearest neighboring BIN (BOLD:AAN0076), which includes specimens of *S.violaceus*, was estimated at 11.44%.

###### Distribution.

Türkiye.

##### ﻿ Sperchon (Hispidosperchon) setiger

Taxon classificationAnimaliaTrombidiformesSperchontidae

Thor, 1898

2CC8A427-E757-5701-BFDC-2C0AF761CBC3

###### Material examined.

**Burdur** • TR23-2024, Çavdır, spring, 37.14478°N, 29.656534°E, 26 Apr. 2024, leg. 1♀ (sequenced) • TR26-2024 Kestel, canal with fast-flowing water, 37.429718°N, 30.399193°E, 27 Apr. 2024, leg. leg. Pešić, Zawal, Gülle & Gülle, 1♀ (sequenced).

###### Remarks.

The sequenced specimens from this study, keyed to *S.setiger* following Di Sabatino et al. (2010), clustered within two unique BINs, i.e. BOLD:AGG3936 and BOLD:AGH7686. The *p*-distance between these two BINs was estimated at 5.97%.

###### Distribution.

Western Palaearctic.

#### ﻿Family Torrenticolidae Piersig, 1902

**Genus *Torrenticola* Piersig, 1896**

##### ﻿ Torrenticola (Torrenticola) baueri

Taxon classificationAnimaliaTrombidiformesTorrenticolidae

Bader & Sepasgozarian, 1987

1E10419E-6EAF-5F76-8273-4C0BDADE3015

###### Material examined.

**Burdur** • TR20-2024, Karacaören, stream, 37.327335°N, 30.869408°E, 25 Apr. 2024, Pešić, Zawal, Gülle & Gülle, 2♂ (sequenced).

###### Remarks.

The sequenced specimens from southwestern Türkiye were clustered within BOLD:AFG4655, which includes specimens from northern Iran and western Türkiye morphologically assigned to *T.baueri*.

###### Distribution.

Iran, Türkiye.

#### ﻿Genus *Monatractides* K. Viets, 1926

##### ﻿ Monatractides (Monatractides) stadleri

Taxon classificationAnimaliaTrombidiformesTorrenticolidae

(Walter, 1924)

CA4DAAA2-4C37-5EDE-87D8-9B70A772ADDE

###### Material examined.

**Burdur** • TR19-2024 waterfall and outflow, 37.33291°N, 30.879221°E, 25 Apr. 2024, leg. Pešić, Zawal, Gülle & Gülle, 1♂ (sequenced), dissected and slide mounted (RMNH).

###### Remarks.

The single male from southwestern Türkiye, molecularly analyzed in this study, matches the description of *M.stadleri*, a species widely distributed in the Mediterranean region, often very frequent in lowland running waters (Di Sabatino et al. 2010). The sequenced specimen was clustered within BOLD:AGC6044 which, in addition to the specimen from this study, includes one specimen from Greece. The *p*-distance to the nearest BINBOLD:AEN9161, which includes specimens of *M.corsicus* from Corsica and Sardinia, was estimated at 8.14%.

In the phylogenetic tree, the sequence obtained from the specimen from Türkiye was nested within clades of *M.stadleri* complex, as a sister clade (albeit with a low support) to *M.corsicus* Pešić & Smit, 2023, a species described from Corsica (Pešić and Smit 2023) and later reported from Sardinia by Pešić and Goldschmidt (2023). The status of the newly detected clade from Türkiye and Greece (BOLD:AGC6044) as well the status of the previously detected clade from Balkans (Montenegro and Greece; BOLD:AEN9161) should be left unnamed until the sequences of *M.stadleri* from its type locality are available.

###### Distribution.

Central, Western, and Southern Europe, Türkiye.

#### ﻿Family Limnesiidae Thor, 1900

**Genus *Limnesia* Koch, 1836**

##### ﻿ Limnesia (Limnesia) fulgida

Taxon classificationAnimaliaTrombidiformesLimnesiidae

Koch, 1836

EDF3D83C-CBA0-5E3D-8097-81937A357AAA

###### Material examined.

**Antalya** • TR27-2024 limnocrene spring, 37.09568°N, 30.58095°E, 27 Apr. 2024, leg. Pešić, Zawal, Gülle & Gülle, 1♂, 2♀ (2♀ sequenced), 1♂ dissected and slide mounted (RMNH).

###### Remarks.

The sequenced specimens from this study identified as *L.fulgida* following Di Sabatino et al. (2010), forms a unique BIN (BOLD:AGG4400). The *p*-distance to its nearest neighboring BIN (BOLD:ACR9738), which includes specimens of *L.fulgida* from the Netherlands and Norway, was estimated at 7.22% suggesting phylogeographic break between European and Turkish populations of this species.

###### Distribution.

Holarctic.

#### ﻿Family Hygrobatidae Koch, 1842

**Genus *Atractides* Koch, 1837**

##### ﻿ Atractides (Atractides) fonticola

Taxon classificationAnimaliaTrombidiformesHygrobatidae

K. Viets, 1920

C6F8CC32-04E0-59C1-B997-3FE0C95AAF51

###### Material examined.

**Burdur** • TR3-2024 Akyayla, rheohelocrenic spring, 37.515774°N, 30.35459°E, 22 Apr. 2024, leg. Pešić, Zawal, Gülle & Gülle, 1♂, 1♀ (sequenced), one palp and I-leg of ♀ dissected and slide mounted. **Isparta** • TR15a-2024 Kışlaköy, spring, 37.66509°N, 30.725111°E, 25 Apr. 2024, leg. Pešić, Zawal, Gülle & Gülle, 1♀ (sequenced).

###### Remarks.

The examined females in our study identified as *A.fonticola* following Gerecke (2003), form a unique BIN (BOLD:AGG3788). The *p*-distance between the latter BIN and its nearest neighbor, BOLD:ADS3489, which includes specimens of *A.fonticola* from Germany, Italy and Montenegro, was estimated at 9.56% suggesting phylogeographic break between European and Turkish populations of this species.

###### Distribution.

Europe, except British Isles and Sweden, Türkiye.

##### ﻿ Atractides (Atractides) graecus

Taxon classificationAnimaliaTrombidiformesHygrobatidae

K. Viets, 1950

B8686C7F-0192-586E-BBC0-1A60A3235320

###### Material examined.

**Burdur** • TR20-2024, Karacaören, stream, 37.327335°N, 30.869408°E, 25 Apr. 2024, Pešić, Zawal, Gülle & Gülle, 1♀ (sequenced), palp and I-leg on one side dissected and slide mounted (RMNH).

###### Remarks.

The single female from Burdur used in this study for molecular analysis matches the description of *A.graecus.* Genetic data indicate that this specimen forms a unique BIN (BOLD:AGG3781).

###### Distribution.

Mediterranean; Türkiye.

##### ﻿ Atractides (Atractides) inflatipalpis

Taxon classificationAnimaliaTrombidiformesHygrobatidae

K. Viets, 1950

8D614F48-D23E-565E-8F36-9EE133BB6E18

Fig. 3

###### Material examined.

**Burdur** • TR3-2024 Akyayla, rheohelocrenic spring, 37.515774°N, 30.35459°E, 22 Apr. 2024, leg. Pešić, Zawal, Gülle & Gülle, 1♂, 2♀ (sequenced), 1♂, 1♀ dissected and slide mounted (RMNH).

###### Remarks.

**Figure 3.**
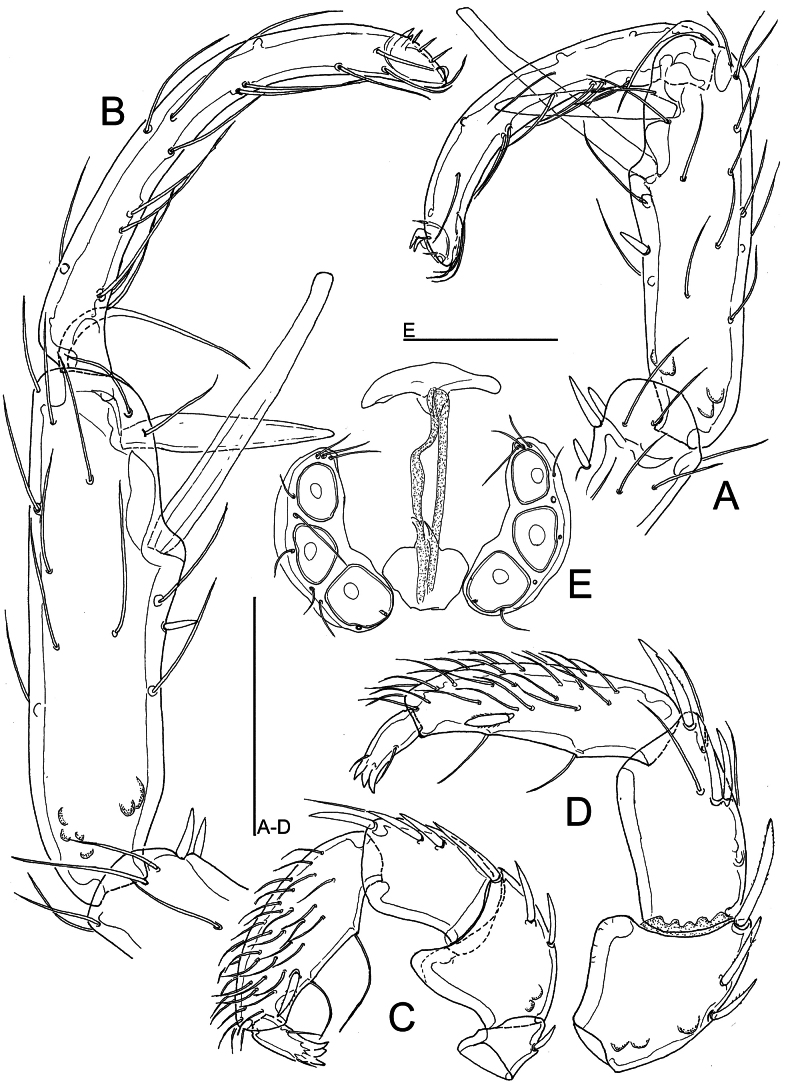
*Atractidesinflatipalpis* K. Viets, 1950 (**A, C** male **B, D–E** female [CCDB-48498-C02], Burdur, Akyayla, rheohelocrenic spring: **A, B** I-L-5 and I-L-6 **C, D** palp, medial view **E** genital field. Scale bars: 100 μm.

In regard to the lineated integument and the shape of I-leg and palp, the examined specimens match the description of *Atractidesinflatipalpis.* These specimens forms a unique BIN (BOLD:AGG3787), with the nearest neighboring BIN being BOLD:AEF1145, which include one female from Montenegro morphologically assigned by the first author to *A.inflatipalpis*. The *p*-distance between these two BINs was estimated at 3.66%. In the phylogenetic tree, the BIN is positioned as a sister clade of *A.fonticolus*. From the latter species, *A.inflatipalpis* differs in more distant S-1 and S-2 setae on I-L-5, a weakly enlarged S-2, a slenderer I-L-6 (Fig. 3A, B) and the strongly protruding finger-like extension of the male P-2 (Fig. 3D).

###### Distribution.

Mediterranean (Greece, Bulgaria, Italy, France, Italy). In Türkiye previously reported from Erzurum Province (Erman et al. 2010).

##### ﻿ Atractides (Atractides) lunipes

Taxon classificationAnimaliaTrombidiformesHygrobatidae

Lundblad, 1956

84AFBE93-A522-59A5-B98A-4121AF50FAA3

###### Material examined.

**Burdur** • TR20-2024, Karacaören, stream, 37.327335°N, 30.869408°E, 25 Apr. 2024, Pešić, Zawal, Gülle & Gülle, 1♀ (sequenced), dissected and slide mounted (RMNH).

###### Remarks.

Genetic data indicate that the examined specimen from Turkey forms a unique BIN (BOLD:AGG3780). The single female from Burdur used in this study for molecular analysis is in good agreement with the redescription of *Atractideslunipes* given by Gerecke (2021) based on populations from Calabria and Sicily. In the phylogenetic tree, the specimen from Türkiye, morphologically assigned to *A.lunipes*, was placed as a sister to *A.zagrosensis* Pesic, Saboori & Asadi, 2016 and *A.corsicus* E. Angelier, 1954, the latter known only from Corsica and Sardinia and considered by Gerecke (2021) as an insular sister species of *A.lunipes*. *Atractideszagrosensis* was originally described by Pesic et al. (2004) as A.cf.lunipes Lundblad, 1956, based on specimens collected in Chahar Mahal and Bakhtiari Province, Iran. Later, this taxon was raised to the status of a separate species (Pešić et al. 2016) and recently barcoded on basis of specimens collected in Iran and Turkey’s Aegean Region (Aydin province, see Pešić et al. 2023a for details). From *Atractideszagrosensis*, *A.lunipes* differs in relatively shorter I-L-5 (I-L-5/6 L ratio 1.15–1.2), more slender setae S-1 and S-2 (L/W S-1 > 17.0, S-2, > 7.0), and slenderer I-L-6 (see Pešić et al. 2016 for details).

###### Distribution.

Western and Southwestern Europe, Türkiye.

##### ﻿ Atractides (Atractides) nikooae

Taxon classificationAnimaliaTrombidiformesHygrobatidae

Pesic, 2004

DE429E7D-79B4-5279-B820-FE36BB150952

###### Material examined.

**Burdur** • TR25-2024 Karamusa stream, 37.186405°N, 29.75374°E, 26 Apr. 2024, leg. Pešić, Zawal, Gülle & Gülle, 2♀ (sequenced) • TR23-2024, Çavdır, spring, 37.14478°N, 29.656534°E, 26 Apr. 2024, leg. Pešić, Zawal, Gülle & Gülle, 1♂ (sequenced), one palp and one I-leg dissected and slide mounted (RMNH).

###### Remarks.

The specimens from southwestern Türkiye match the description of *Atractidesnikooae*, a species originally described from the Markazi Province, West Iran (Pesic et al. 2004), and later reported from Siirt Province in Turkey (Esen et al. 2013a). The specimens from Türkiye form a unique BIN (BOLD:AGG3766), with the nearest neighboring BIN being BOLD:ACS0163, which includes specimens of *A.distans* from the Netherlands, Poland, and Italy. The *p*-distance between these two BINs is estimated at 14.63%.

###### Distribution.

Iran, Türkiye.

##### ﻿ Atractides (Atractides) robustus

Taxon classificationAnimaliaTrombidiformesHygrobatidae

(Sokolow, 1940)

292FB36C-FD35-521D-9C4E-19A40A292461

###### Material examined.

**Isparta** • TR17-2024 Çukurköy, stream, 37.651257°N, 30.81791°E, 25 Apr. 2024, leg. 1♂ (sequenced) • TR18-2024, Yazılıkanyon Tabiat Parkı, stream from cave (moss), 37.46882°N, 30.919449°E, 25 Apr. 2024, leg. Pešić, Zawal, Gülle & Gülle, 1deutonymph (sequenced).

###### Remarks.

The sequenced specimens from southwestern Türkiye were clustered within two BINs, i.e. BOLD:AGH5609 which include one specimen from this study and BOLD:AEK3669, which in addition to a single deutonymph from our study includes one specimen of *A.robustus* from eastern Türkiye (Bingöl province). The *p*-distance between these two BINs was estimated at 2.89%.

For a long time, *A.robustus*, a species originally described from the Caucasus (the affluents of the Kuban River) has been considered as a common species in Europe (Gerecke 2003). However, recent genetic data revealed that this species consists of several distinct lineages (Pešić et al. 2023b). The results of the applied ASAP procedure revealed that both BINs from southwestern Türkiye belong to a single MOTU (hypothetical species), which grouped also sequences of *A.robustus* like mites from eastern Türkiye and northern Iran. On the other hand, the second MOTU, which includes European populations of *robustus*-like mites belonging to BOLD:ADZ9348 and BOLD:AFF2463, possible represent a cryptic species new to science.

###### Distribution.

Europe, Türkiye, Caucasus, Iran.

##### ﻿ Atractides (Atractides) subasper

Taxon classificationAnimaliaTrombidiformesHygrobatidae

Koenike, 1902

06B1FD90-60A7-5768-86E0-3AF112C9F4B8

###### Material examined.

**Burdur** • TR11-2024, Söbüce, first order stream, 37.287872°N, 30.067743°E, 24 Apr. 2024, leg. Pešić, Zawal, Gülle & Gülle, 1♀ (sequenced), dissected and slide mounted (RMNH).

###### Remarks.

The female from Burdur used in this study for molecular analysis, matches the description of *A.subasper*, a species easily identified by a pointed and protruding gnathosomal rostrum, three pairs of acetabula arranged in a weakly curved line and a rather homomorphic S-1 and S-2 setae on I-L-5 (see Gerecke 2003). The specimen from Türkiye forms a unique BIN (BOLD:AGG3778) with the nearest neighboring BIN being BOLD:AEX4044, which includes specimens from Serbia, Italy, Bulgaria, Albania, and Switzerland. The *p*-distance between these two BINs is estimated at 8.67%. The only public sequence in BOLD:AEX4044 belongs to a female specimen from Serbia morphologically assigned by Jovanović et al. (2024) to *A.glandulosus* (Walter, 1918), a species with certainty known from the Alps, southern Germany, and the Pyrenees (Gerecke 2003). Re-examination of the female from Serbia revealed good agreement with *A.subasper*, except in the shape of the gnathosoma, which is with a short rostrum, not pointed and protruding as in typical *subasper* specimens. The results of the applied ASAP procedure grouped the sequences of these two BINS into the same MOTU (hypothetical species). The BINBOLD:AES6460 which groups the sequence of *A.subasper* from Sardinia forms a separate MOTU with a high genetic distance (10.91% *p*-distance) to the next closest BIN of *A.subasper* (BOLD:AEX4044) indicating that the populations from Sardinia probably represent a species new to science.

###### Distribution.

Central and southern Europe, Türkiye, Caucasus.

##### ﻿ Atractides (Atractides) turani

Taxon classificationAnimaliaTrombidiformesHygrobatidae

Pešić, Zawal, Gülle & Smit sp. nov.

10E5FFC8-403F-5174-A73F-95878511F64F

https://zoobank.org/260B41B7-7CE4-429A-BBD3-CD585188C062

Fig. 4

###### Type material.

***Holotype*** • ♂, sequenced (Voucher Id: CCDB-48498-A09), dissected and slide mounted (RMNH), Burdur, TR11-2024, Söbüce, stream, 37.287872°N, 30.067743°E, 24 Apr. 2024, leg. Pešić, Zawal, Gülle & Gülle.

###### Other material.

• 1♂, sequenced (Voucher Id: CCDB-48498-F11), one palp and one I-leg dissected and conserved in Koenike’s fluid, **Burdur**, TR2-2024 Akyayla, rheocrenic spring, 37.482956°N, 30.326647°E, 22 Apr. 2024, leg. Pešić, Zawal, Gülle & Gülle.

###### Diagnosis.

(female unknown) Integument lineated, P-2 with a weakly developed protrusion, P-4 sword seta slightly posterior to distoventral seta, the latter closely approaching the distoventral edge of the segment, I-L-5 elongate, with setae S-1/-2 close to each other, interspace 9 µm, I-L-6 shortened and distally narrowed, L I-L-5/6 ratio > 1.6.

###### Description.

**Male** (holotype). Integument lineated; dorsal and ventrocaudal idiosoma without sclerotized muscle insertions. Genital plate with nearly straight anterior margin, posterior margin medially indented in an obtuse angle. Gonopore long, flanked by ~ 10 pairs of fine setae. Acetabula subtriangular, arranged in an obtuse triangle. Excretory pore smooth, Vgl-1/2 separate.

**Figure 4.**
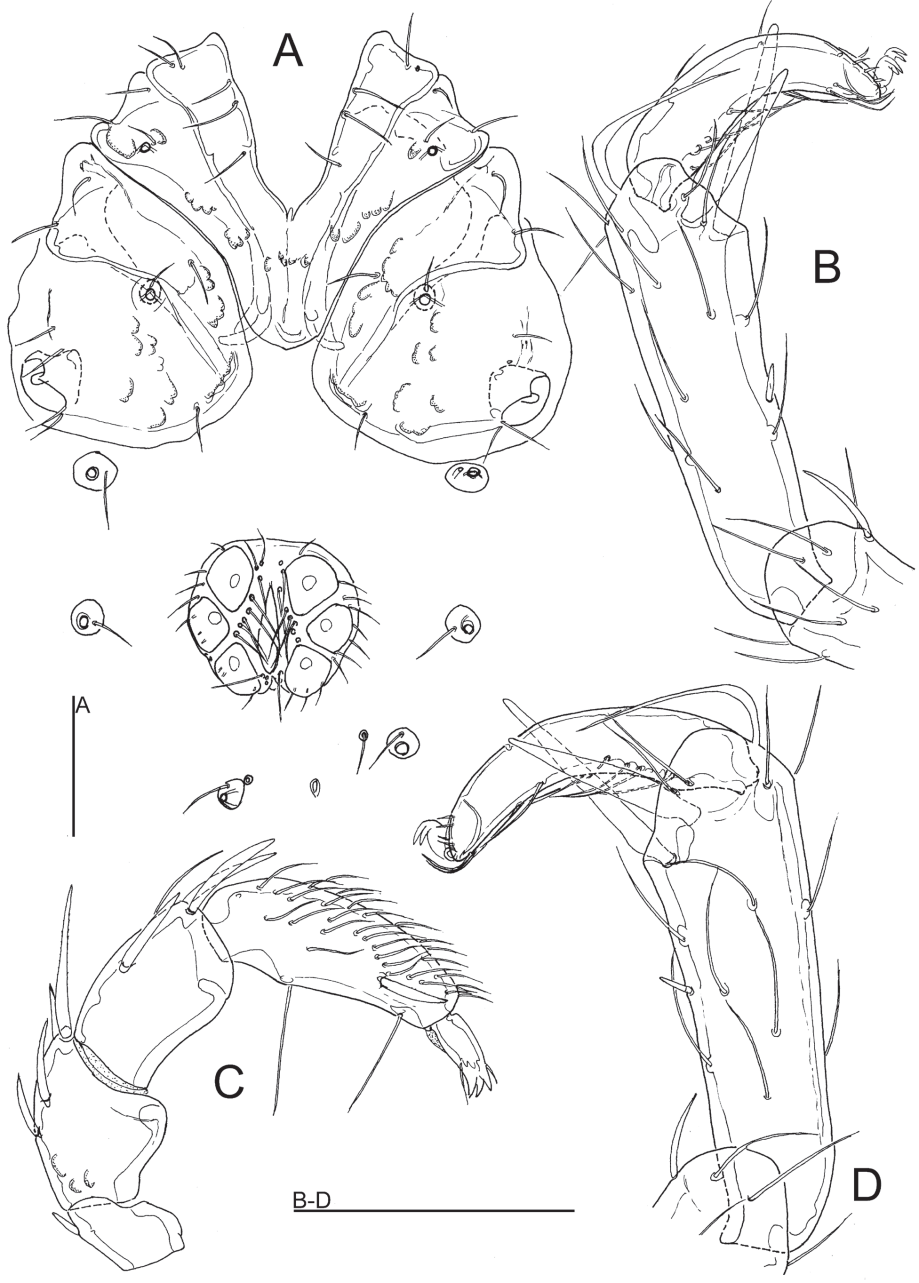
*Atractidesturani* sp. nov., ♂ (**A–C** holotype [CCDB-48498-A09] **D** specimen from Akyayla [CCDB-48498-F11], Burdur, Söbüce, stream: **A** coxal and genital field **B, D** I-L-5 and I-L-6 **C** palp, medial view. Scale bars: 100 μm.

Gnathosoma without particularly projecting rostrum, palp with a rounded distoventral projection at P-2, P-4 slightly protruding near proximoventral seta, sword seta long and curved, inserting slightly posterior to distoventral seta, distoventral seta closely approaching the distoventral edge of the segment (Fig. 4C), ventral margin divided in sectors 3: 5: 1.

I-L-5 elongate, dorsal and ventral margin diverging only towards distal segment end; setae S-1 and S-2 bluntly pointed and close to each other (separation < 10 µm), S-1 slightly shorter and more slender than S-2; I-L-6 shortened and curved, equally narrowed from base to tip (Fig. 4B).

***Measurements*** (holotype [CCDB-48498-A09], in parentheses some measurements of specimen from Akyayla [CCDB-48498-F11] — Idiosoma L 590 (603), W (456); maximum diameter Dgl-4, 24 (25). Coxal shield L 303; Cx-III W 344; Cx-I+II mL 94, Cx-I+II lL 211. Genital field L/W 116 (127)/125 (132), ratio 0.93 (0.96), L Ac-1-3: 44 (45–48), 39–42 (48–56), 38–39 (42).

Palp — Total L 288 (307); dL/H: P-1, 30/30 (33/30); P-2, 66/48 (69/53); P-3, 64/42 (66/42); P-4, 97/37 (105/35); P-5, 31/12 (34/12); L ratio P-2/P-4, 0.68 (0.63). Chelicera total L 197, capitulum vL 121.

Legs — I-L-5 dL 170 (183), vL 134 (138), dL/vL ratio 1.28 (1.33), maximum H 50 (58), dL/maximum H ratio 3.4 (3.16), S-1 L 77 (88), L/W ratio 9.2 (10.4), S-2 L 65 (73), L/W ratio 5.5 (6.7), distance S-1-2, 9 (11), dL ratio S-1/2, 1.19 (1.14); I-L-6 dL 95 (113), central H 22 (23), dL/central H ratio 4.27 (4.9); L I-L-5/6 ratio 1.79 (1.62).

**Female.** Unknown.

###### Etymology.

The new species is named after Prof. Davut Turan (Recep Tayyip Erdoğan University, Rize, Türkiye) in appreciation of his comprehensive work on Turkish ichthyofauna.

###### Species delimitation using DNA barcodes.

**Figure 5.**
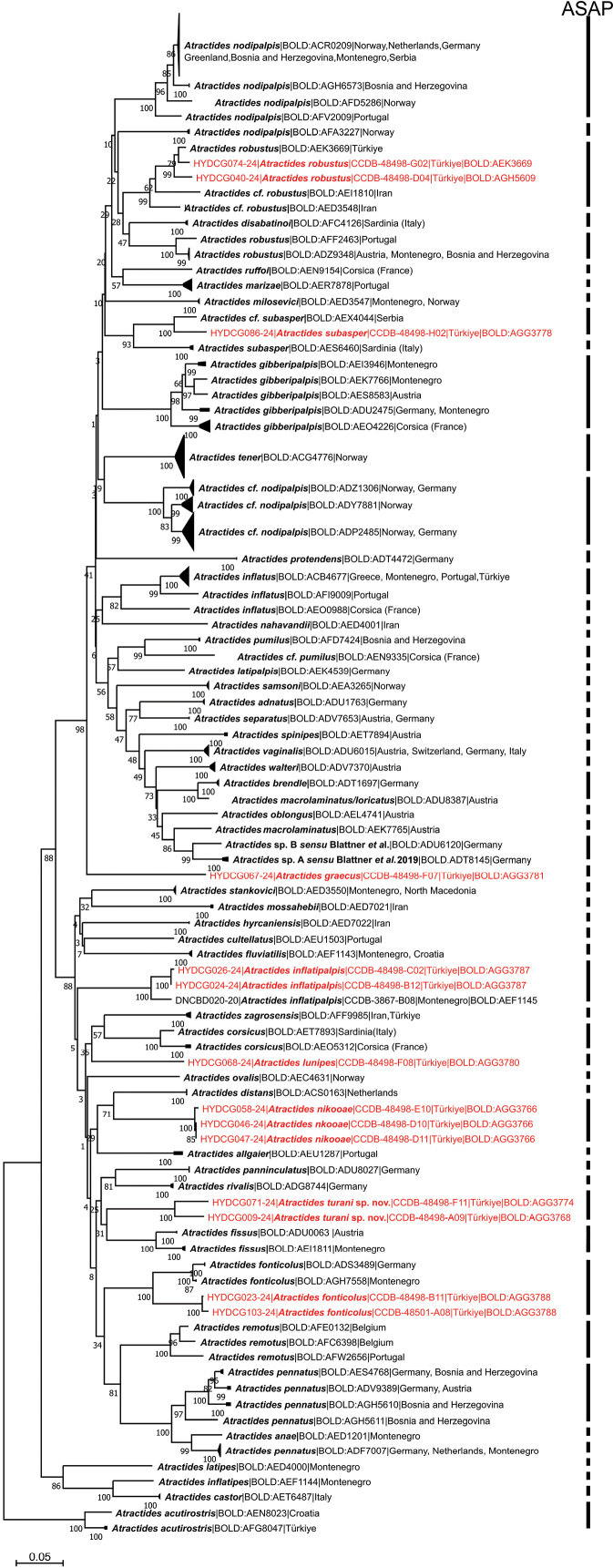
Neighbor-Joining tree of the subggenus *Atractides* s. str. obtained from 353 nucleotide COI sequences listed in Suppl. material 1. *Atractidesacutirostris* (Motas & Angelier, 1927) from the subgenus Tympanomegapus was used to root the tree. The results of species delimitation by ASAP procedure are indicated by vertical bars. BINs are based on the barcode analysis from 4 November 2024. New sequences from this study are marked in red.

The final alignment for species delimitation using COI sequence data comprised 681 nucleotide positions (nps) of the 353 *Atractides* specimens listed in Suppl. material 1. *Atractidesacutirostris* from the subgenus Tympanomegapus was used to root the tree. The NJ tree is presented in Fig. 5. The COI tree sequences retrieved from two specimens collected in southeastern Türkiye reveals the presence of two maximally supported clades corresponding to each of sequenced individuals. These two clades were placed, albeit with a low support, as a sister group to the cluster of sequences belonging to *A.fissus* (Walter, 1927).

###### Differential diagnosis.

In the Western Palaearctic fauna, the most similar species (combining a lineated integument with a weakly developed protrusion of P-2 in males) are *A.panniculatus* (K. Viets, 1925), *A.fissus* (Walter, 1927), *A.rivalis* Lundblad, 1956, and *A.elazigensis* Esen & Pešić, 2024. From all these species (in parentheses data from Gerecke 2003 and Esen and Pešić 2024), the new species can be distinguished by having a narrower separation of setae S-1/2, < 10 µm (> 20 µm), a rather high I-L-5/6 ratio of 1.7 (L ratio I-L-5/6 < 1.5) and I-L-6 basally thickened and distally narrowed (I-L-6 longer, not narrowed in distal part).

Further European species with a rather high I-L-5/6 ratio (for details see Gerecke 2003) are *Atractidesfonticolus* (K. Viets, 1920), *A.pennatus* (K. Viets, 1920), and *A.legeri* (Motaș, 1927). These three species form a distinctive species group, differing from the new species in the shape of P-2 with a ventral projection in males and the shape of I-L-6 (with dorsal and ventral margins parallel up to the base of the claw furrow, distally not continuously narrowed, see Gerecke 2003 for details).

The single sequenced male collected in spring near Akyayla differs from the type specimen in longer and consequently more slender I-L-6 (compare Fig. 4B with Fig. 4C). Genetic data indicate that this specimen forms a unique BIN (BOLD:AGG3774). The *p*-distance between this BIN and BOLD:AGG3768, which include type specimen of *A.turani* sp. nov., was estimated at 6.26%. The results of the applied ASAP procedure grouped the sequences of these two BINS into the same MOTU (hypothetical species).

###### Distribution.

Türkiye.

#### ﻿Genus *Hygrobates* Koch, 1837

##### ﻿ Hygrobates (Hygrobates) persicus

Taxon classificationAnimaliaTrombidiformesHygrobatidae

Pešić & Asadi, 2017

E1BA4479-0EEA-5CE5-BAAB-5DDEE537CBFE

###### Material examined.

**Antalya** • TR28-2024 Düden river, 36.959763°N, 30.731194°E, 27 Apr. 2024, leg. leg. Pešić, Zawal, Gülle & Gülle, 1♂, 3♀ (sequenced). **Isparta** • TR15a-2024 Kışlaköy, spring, 37.66509°N, 30.725111°E, 25 Apr. 2024, leg. Pešić, Zawal, Gülle & Gülle, 2♂ (sequenced).

###### Remarks.

The specimens from southwestern Türkiye morphologically match the description of *Hygrobatespersicus*. This species is widely distributed in Iran, while in Türkiye it is known only from the Çoruh River in northeast Anatolia (Bayburt province, Pešić et al. 2017). The sequenced specimens from Antalya and Isparta provinces cluster within BOLD:ACB5533, which, in addition to the specimens used in this study, includes specimens morphologically assigned to *H.persicus*.

###### Distribution.

Iran, Türkiye.

##### ﻿ Hygrobates (Hygrobates) longipalpis

Taxon classificationAnimaliaTrombidiformesHygrobatidae

(Hermann, 1804)

BA5B4538-B7E0-5BC9-80B6-9FAB1E8FDF34

###### Material examined.

**Burdur** • TR6-2024 Sazak, rheocrenic spring, 37.544933°N, 29.94381°E, 23 Apr. 2024, leg. 1♂, 1♀ (sequenced) • TR12-2024, Burdur, Kayalı, limnocrene spring, 37.306606°N, 29.931082°E, 24 Apr. 2024, leg. Pešić, Zawal, Gülle & Gülle, 1♂ (sequenced) • TR14-2024 Dereköy, spring, 37.42846°N, 29.809637°E, 24 Apr. 2024, leg. Pešić, Zawal, Gülle & Gülle, 1♀ (sequenced). **Isparta** • TR15-2024, Kışlaköy, river, 37.66509°N, 30.725111°E, 25 Apr. 2024, leg. Pešić, Zawal, Gülle & Gülle, 1♂ (sequenced). **Antalya** • TR27-2024 limnocrene spring, 37.09568°N, 30.58095°E, 27 Apr. 2024, leg. Pešić, Zawal, Gülle & Gülle, 1♂ (sequenced).

###### Remarks.

The sequenced specimens from this study are clustered in BOLD:AES0232, which, in addition to the specimens from this study, includes specimens from North Macedonia and Montenegro as well as specimens from Burdur province identified as *H.longipalpis* in Pešić et al. (2023c).

###### Distribution.

Western Palaearctic.

##### ﻿ Hygrobates (Rivobates) quanaticola

Taxon classificationAnimaliaTrombidiformesHygrobatidae

Schwoerbel & Sepasgozarian, 1976

25C8849E-2710-5D8B-B35C-DE04E292DD52

###### Material examined.

**Burdur** • TR1-2024 Kuzköy, spring, 37.55402°N, 30.440313°E, 22 Apr. 2024, leg. Pešić, Zawal, Gülle & Gülle 3♀ (sequenced) • TR14-2024 Dereköy, rheocrenic spring, 37.42846°N, 29.809637°E, 24 Apr. 2024, leg. Pešić, Zawal, Gülle & Gülle, 1♀ (sequenced) • TR26-2024 Kestel, canal with fast-flowing water, 37.429718°N, 30.399193°E, 27 Apr. 2024, leg. Pešić, Zawal, Gülle & Gülle, 1♀ (sequenced).

###### Remarks.

The sequences obtained from the specimens assigned morphologically to *H.quanaticola* are clustered within two BINs, i.e. BOLD:AGG3789, which includes three specimens from this study, and BOLD:AEM9575, which in addition to two specimens from this study, includes three specimens from Burdur published in Pešić et al. (2023c). The *p*-distance between these two BINs was estimated at 2.57%.

###### Distribution.

Iran, Türkiye.

#### ﻿Family Unionicolidae Oudemans, 1909

**Subfamily Pionatacinae K. Viets, 1916**

**Genus *Neumania* Lebert, 1879**

##### ﻿ Neumania (Neumania) imitata

Taxon classificationAnimaliaTrombidiformesUnionicolidae

Koenike, 1908

19E4B78A-B0F9-565A-966E-A66E6230FD4D

###### Material examined.

**Antalya** • TR29-2024 Aksu, pond near Antalya city, 36.87547°N, 30.8454°E, 27 Apr. 2024 leg. Pešić, Zawal, Gülle & Gülle, 2♀ (sequenced).

###### Other material examined.

*Neumaniaimitata*, the Netherlands, Merkske • Halsche Beemden, 51.422°N, 4.826°E, 13 Jun. 2016 leg. Smit 3♂ (sequenced; NLACA976-17/RMNH.5070734, NLACA977-17/ RMNH.5070735, NLACA978-17/ RMNH.5070736), dissected and slide mounted (RMNH).

###### Remarks.

The examined male in our study, identified as *Neumaniaimitata* following Gerecke et al. (2016), forms a unique BIN (BOLD:AGG4333). The *p*-distance between this BIN and its nearest neighbor, BOLD:ADF7924, which includes specimens of *N.imitata* from the Netherlands, is estimated at 2.25%.

###### Distribution.

Europe; rare, reported from France, Italy, Germany, the Netherlands, Poland, Montenegro, and Portugal (Pešić et al. 2024).

##### ﻿ Neumania (Neumania) limosa

Taxon classificationAnimaliaTrombidiformesUnionicolidae

(Koch, 1836)

4AB7E333-8160-5033-A255-DBA6DE0B0C45

###### Material examined.

**Burdur** • TR5-2024 Düger, limnocrene spring, 37.574345°N, 30.021276°E, 23 Apr. 2024, leg. Pešić, Zawal, Gülle & Gülle, ♀ (sequenced).

###### Remarks.

The sequenced specimens from this study cluster in BOLD:AEF5902, which includes specimens of *N.limosa* from Montenegro. The *p*-distance between this BIN and its nearest neighbor BOLD:ACS0551, which includes specimens from the Netherlands and Portugal assigned to *N.limosa*, is estimated at 3.21%.

###### Distribution.

Palaearctic.

#### ﻿Family Pionidae Thor, 1900

**Genus *Piona* Koch, 1842**

##### ﻿ Piona alpicola

Taxon classificationAnimaliaTrombidiformesPionidae

(Neuman, 1880)

144A02DA-7495-52CE-8B8D-413EAC4A0B27

###### Material examined.

**Burdur** • TR22-2024 Uylupınar, limnocrene spring, 37.10993°N, 29.613293°E, 26 Apr. 2024, leg. Pešić, Zawal, Gülle & Gülle, 1♀ (sequenced).

###### Remarks.

The single female from Burdur, molecularly analyzed in this study, clusters in BOLD:ACR9570, which includes specimens from the Netherlands.

###### Distribution.

Holarctic.

#### ﻿Family Arrenuridae Thor, 1900

**Genus *Arrenurus* Dugès, 1834**

##### ﻿ Arrenurus (Arrenurus) compactus

Taxon classificationAnimaliaTrombidiformesArrenuridae

Piersig, 1894

95D98BC9-566E-56FD-A8D5-4F02AC666AE8

###### Material examined.

**Burdur** • TR22-2024 Uylupınar, limnocrene spring, 37.10993°N, 29.613293°E, 26 Apr. 2024, leg. Pešić, Zawal, Gülle & Gülle, 1♀ (sequenced).

###### Remarks.

The examined female from Burdur clusters in BOLD:AEJ6492, which includes a single specimen from Norway assigned to *Arrenuruscompactus*.

###### Distribution.

Palaearctic.

##### ﻿ Arrenurus (Arrenurus) suecicus

Taxon classificationAnimaliaTrombidiformesArrenuridae

Lundblad, 1917

5738299C-DC77-5D55-890E-3F266FE45210

###### Material examined.

**Burdur** • Uylupinar, limnocrene spring, 37.10993°N, 29.613293°E, 26 Apr. 2024, leg. Pešić, Zawal, Gülle & Gülle, 1♀ (sequenced).

###### Remarks.

The examined female from Burdur clusters in BOLD:AAV9863, which includes a specimen of *Arrenurussuecicus* from Sweden. The *p*-distance from the nearest neighboring BINBOLD:ADF6369, which includes a single specimen of *A.suecicus* from the Netherlands, was estimated at 2.24%.

###### Distribution.

Western Palaearctic.

##### ﻿ Arrenurus (Truncaturus) fontinalis

Taxon classificationAnimaliaTrombidiformesArrenuridae

K. Viets, 1920

21D6F26C-4B1B-5EFD-B5B1-CCB59D49703D

###### Material examined.

**Burdur** • TR3-2024 Akyayla, rheohelocrenic spring, 37.515774°N, 30.35459°E, 22 Apr. 2024, leg. Pešić, Zawal, Gülle & Gülle, 2♀ (sequenced) • TR9 Kemer, helocrenic spring 37.301468°N, 30.097061°E, 22 Apr. 2024, leg. Pešić, Zawal, Gülle & Gülle, 1♂, 2♀ (sequenced).

**Figure 6.**
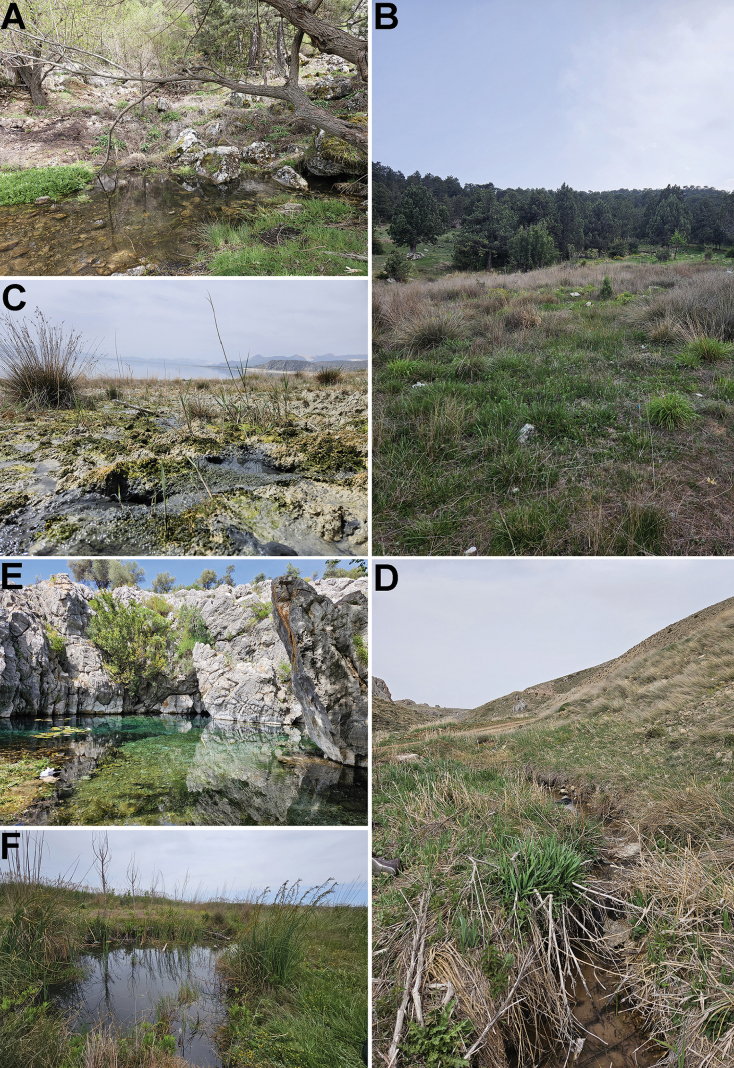
Photographs of selected sampling sites **A** Burdur, TR2-2024 Akyayla, spring **B** Burdur, TR3-2024 Akyayla, rheohelocrenic spring **C** Burdur, TR4-2024 helocrenic spring near Burdur Lake **D** Burdur, TR11-2024, Söbüce, first order stream **E** Antalya, TR27-2024 limnocrene spring **F** Antalya, TR29-2024 Aksu, pond. Photographs by VP.

###### Remarks.

The sequenced specimens from southwestern Türkiye form a unique BIN (BOLD:AGH5781) with the nearest neighboring BIN being BOLD:ADS8719, which includes two specimens of *A.fontinalis*, one from the Germany and one from an unknown locality. The *p*-distance between these two BINs was estimated at 11.78%, indicating the need for taxonomic revision of *A.fontinalis* complex to identify possible undescribed cryptic species.

###### Distribution.

Western Palaearctic.

## ﻿Conclusions

This study provides the first insight into the molecular diversity of water mites in southwestern Türkiye. The formation of a DNA barcode reference library, one of the key results of this study, is a continuation of our ongoing work on the molecular characterization of water mites that inhabit Türkiye. BOLD contains a relatively small number of barcodes of Turkish water mites, with approximately 44% of the barcodes added by this study. Despite the short collection period and the limited number of individuals included in the molecular analyses, our study provided 40 BINs, 23 of which were new to BOLD. Broader sampling during different seasons and more comprehensive efforts through various ongoing barcode initiatives at the regional and international level would certainly result in higher estimates of the molecular richness of water mites in the study area.

## Supplementary Material

XML Treatment for Hydrachna globosa

XML Treatment for Hydrodroma torrenticola

XML Treatment for Protzia longiacetabulata

XML Treatment for Protzia vietsi

XML Treatment for Iranothyas marismortui

XML Treatment for Trichothyas (Lundbladia) petrophila

XML Treatment for Lebertia (Lebertia) glabra

XML Treatment for Lebertia (Lebertia) rivulorum

XML Treatment for Sperchon beneckei

XML Treatment for Sperchon (Hispidosperchon) compactilis

XML Treatment for Sperchon (Sperchon) thienemanni

XML Treatment for Sperchon (Hispidosperchon) papillosus

XML Treatment for Sperchon (Hispidosperchon) serapae

XML Treatment for Sperchon (Hispidosperchon) setiger

XML Treatment for Torrenticola (Torrenticola) baueri

XML Treatment for Monatractides (Monatractides) stadleri

XML Treatment for Limnesia (Limnesia) fulgida

XML Treatment for Atractides (Atractides) fonticola

XML Treatment for Atractides (Atractides) graecus

XML Treatment for Atractides (Atractides) inflatipalpis

XML Treatment for Atractides (Atractides) lunipes

XML Treatment for Atractides (Atractides) nikooae

XML Treatment for Atractides (Atractides) robustus

XML Treatment for Atractides (Atractides) subasper

XML Treatment for Atractides (Atractides) turani

XML Treatment for Hygrobates (Hygrobates) persicus

XML Treatment for Hygrobates (Hygrobates) longipalpis

XML Treatment for Hygrobates (Rivobates) quanaticola

XML Treatment for Neumania (Neumania) imitata

XML Treatment for Neumania (Neumania) limosa

XML Treatment for Piona alpicola

XML Treatment for Arrenurus (Arrenurus) compactus

XML Treatment for Arrenurus (Arrenurus) suecicus

XML Treatment for Arrenurus (Truncaturus) fontinalis

## ﻿Additional information

### Author ORCIDs

Vladimir Pešić https://orcid.org/0000-0002-9724-345X

Andrzej Zawal https://orcid.org/0000-0002-5838-6060

Milica Jovanović https://orcid.org/0000-0003-1836-1210

Aleksandra Bańkowska https://orcid.org/0000-0003-1037-0550

Stanisław Musielak https://orcid.org/0000-0002-4770-1432

Harry Smit https://orcid.org/0000-0002-0376-6808

### Data availability

All of the data that support the findings of this study are available in the main text or Supplementary Information.

## Supplementary materials

Supplementary material 1 List of *Atractides* and *Sperchon* specimens Data type docx Explanation note List of specimens of genera *Sperchon* and *Atractides*, respectively, used for building the Neighbor-Joining (NJ) tree (Figs 2, 5). https://binary.pensoft.net/file/1284174This dataset is made available under the Open Database License (http://opendatacommons.org/licenses/odbl/1.0/). The Open Database License (ODbL) is a license agreement intended to allow users to freely share, modify, and use this Dataset while maintaining this same freedom for others, provided that the original source and author(s) are credited.Vladimir Pešić, Andrzej Zawal, Pınar Gülle, İskender Gülle, Milica Jovanović, Aleksandra Bańkowska, Stanisław Musielak, Harry Smit
