# Supplementary material for: ﻿Water mite diversity from southwestern Türkiye through the lens of the DNA barcodes, with the description of one new species (Acari, Hydrachnidia)
Source: Zookeys. 2025 Mar 18;1232:205–36. doi: 10.3897/zookeys.1232.142699 (PMC11937902; doi:10.3897/zookeys.1232.142699)
Supplement: Supplementary material 1 — List of Atractides and Sperchon specimens [file zookeys-1232-205_article-142699__-s001.docx]

**Supplementary material.** List of *Sperchon and Atractides* specimens, respectively, used for building the Neighbour-Joining (NJ) tree.

List of *Sperchon* specimens used for building the Neighbour-Joining (NJ) tree (Fig. 2).

| *Sperchon brevirostris* | HYDCA098-16 | HYDCA98 | Germany | BOLD:ACP6107 |  |
| --- | --- | --- | --- | --- | --- |
|  | HYDCA097-16 | HYDCA97 | Germany |  |  |
|  | HYDCA096-16 | HYDCA96 | Germany |  |  |
|  | MARBN820-23 | MARB UIB 1093 | Norway |  |  |
|  | HYDCA121-18 | HYDCA121 | Norway |  |  |
|  | MARBN730-23 | MARB UIB 1193 | Norway |  |  |
|  | MARBN1178-23 | MARB UIB 786 | Norway |  |  |
|  | MARBN965-23 | MARB UIB 953 | Norway |  |  |
|  | MARBN939-23 | MARB UIB 1307 | Norway |  |  |
|  | MARBN923-23 | MARB UIB 1291 | Norway |  |  |
|  | MARBN826-23 | MARB UIB 1099 | Norway |  |  |
|  | MARBN819-23 | MARB UIB 1092 | Norway |  |  |
|  | MARBN769-23 | MARB UIB 1042 | Norway |  |  |
|  | MARBN768-23 | MARB UIB 1041 | Norway |  |  |
|  | MARBN731-23 | MARB UIB 1194 | Norway |  |  |
|  | MARBN692-23 | MARB UIB 1155 | Norway |  |  |
|  | MARBN691-23 | MARB UIB 1154 | Norway |  |  |
|  | MARBN681-23 | MARB UIB 1144 | Norway |  |  |
|  | MARBN680-23 | MARB UIB 1143 | Norway |  |  |
|  | MARBN851-23 | MARB UIB 1124 | Norway |  |  |
|  | MARBN688-23 | MARB UIB 1151 | Norway |  |  |
|  | MARBN884-23 | MARB UIB 1252 | Norway |  |  |
|  | MARBN885-23 | MARB UIB 1253 | Norway |  |  |
|  | MARBN825-23 | MARB UIB 1098 | Norway |  |  |
|  | EBAHY006-16 | EBAI-Hyd06 | Norway |  |  |
|  | EBAHY005-16 | EBAI-Hyd05 | Norway |  |  |
|  | EBAHY004-16 | EBAI-Hyd04 | Norway |  |  |
|  | EBAHY002-16\| | EBAI-Hyd02 | Norway |  |  |
|  | EBAHY001-16 | EBAI-Hyd01 | Norway |  |  |
|  | EBAHY003-16 | EBAI-Hyd03 | Norway |  |  |
|  | HYDCA113-18 | HYDCA113 | Norway |  |  |
|  | MARBN766-23 | MARB UIB 1039 | Norway | BOLD:AFD0340 |  |
|  | MARBN1179-23 | MARB UIB 787 | Norway |  |  |
|  | MARBN922-23 | MARB UIB 1290 | Norway |  |  |
|  | HYDCA099-16 | HYDCA99 | Germany |  |  |
|  | DNAEC018-20 | 8. ME2019 4 B7 | North Macedonia | BOLD:AED3857 |  |
|  | DCCDB011-21 | CCDB38233 A11 | Montenegro |  |  |
| *Sperchon cf. glandulosus*  (Norway) | EBAHY010-16 | EBAI-Hyd10 | Norway | BOLD:ADC0986 |  |
|  | EBAHY011-16 | EBAI-Hyd11 | Norway |  |  |
| *Sperchon glandulosus* | *ACHAR465-18 | MN670825 | Canada | BOLD:ACR5909 |  |
|  | *ACHAR466-18 | MN673973 | Canada |  |  |
|  | *ACHAR464-18 | MN668480 | Canada |  |  |
|  | *ACHAR467-18 | MN670917 | Canada |  |  |
|  | *ABOTH11463-23 |  | Canada |  |  |
|  | *ABOTH11458-23 |  | Canada |  |  |
|  | HYDCA122-18 | HYDCA122 | Norway |  |  |
|  | MARBN1325-23 | MARB UIB 933 | Norway |  |  |
|  | MARBN1181-23 | MARB UIB 789 | Norway |  |  |
|  | MARBN1182-23 | MARB UIB 790 | Norway |  |  |
|  | MARBN839-23 | MARB UIB 1112 | Norway | BOLD:ACQ0530 |  |
|  | MARBN771-23 | MARB UIB 1044 | Norway |  |  |
|  | MARBN982-23 | MARB UIB 970 | Norway |  |  |
|  | MARBN633-23 | MARB UIB 716 | Norway |  |  |
|  | MARBN632-23 | MARB UIB 715\| | Norway |  |  |
|  | MARBN1266-23 | MARB UIB 874 | Norway |  |  |
|  | MARBN671-23 | MARB UIB 1134 | Norway |  |  |
|  | MARBN770-23 | MARB UIB 1043 | Norway |  |  |
|  | MARBN1077-23 | MARB UIB 590 | Norway |  |  |
|  | MARBN601-23 | MARB UIB 684 | Norway |  |  |
|  | MARBN1131-23 | MARB UIB 644 | Norway |  |  |
|  | MARBN574-23 | MARB UIB 657 | Norway |  |  |
|  | MARBN915-23 | MARB UIB 1283 | Norway |  |  |
|  | MARBN1108-23 | MARB UIB 621 | Norway |  |  |
|  | MARBN1323-23 | MARB UIB 931 | Norway |  |  |
|  | MARBN1324-23 | MARB UIB 932 | Norway |  |  |
|  | MARBN733-23 | MARB UIB 1196 | Norway |  |  |
|  | MARBN916-23 | MARB UIB 1284 | Norway |  |  |
|  | MARBN966-23 | MARB UIB 954 | Norway |  |  |
|  | MARBN1132-23 | MARB UIB 645 | Norway |  |  |
|  | MARBN1030-23 | MARB UIB 1018 | Norway |  |  |
|  | MARBN1290-23 | MARB UIB 898 | Norway |  |  |
|  | MARBN1191-23 | MARB UIB 799 | Norway |  |  |
|  | MARBN599-23 | MARB UIB 682 | Norway |  |  |
|  | MARBN1100-23 | MARB UIB 613 | Norway |  |  |
|  | MARBN689-23 | MARB UIB 1152 | Norway |  |  |
|  | MARBN765-23 | MARB UIB 1038 | Norway |  |  |
|  | MARBN1326-23 | MARB UIB 934 | Norway |  |  |
|  | MARBN1267-23 | MARB UIB 875 | Norway |  |  |
|  | MMHYD061-19 | HYDCA195 | Norway |  |  |
|  | MARBN1289-23 | MARB UIB 897 | Norway |  |  |
|  | MARBN1137-23 | MARB UIB 650 | Norway |  |  |
|  | MARBN1099-23 | MARB UIB 612 | Norway |  |  |
|  | MARBN1020-23 | MARB UIB 1008 | Norway |  |  |
|  | MARBN732-23 | MARB UIB 1195 | Norway |  |  |
|  | MMHYD387-21 | HYDCA521 | Norway |  |  |
|  | MARBN687-23 | MARB UIB 115 | Norway |  |  |
|  | MARBN929-23 | MARB UIB 1297 | Norway |  |  |
|  | MARBN614-23 | MARB UIB 697 | Norway |  |  |
|  | MARBN918-23 | MARB UIB 1286 | Norway |  |  |
|  | MARBN1029-23 | MARB UIB 1017 | Norway |  |  |
|  | MARBN1008-23 | MARB UIB 996 | Norway |  |  |
|  | MARBN690-23 | MARB UIB 1153 | Norway |  |  |
|  | MARBN862-23 | MARB UIB 1230 | Norway |  |  |
|  | MARBN1004-23 | MARB UIB 992 | Norway |  |  |
|  | MARBN635-23 | MARB UIB 718 | Norway |  |  |
|  | EBAHY007-16 | EBAI-Hyd07 | Norway |  |  |
|  | MARBN602-23 | MARB UIB 685 | Norway |  |  |
|  | MARBN914-23 | MARB UIB 1282 | Norway |  |  |
|  | MARBN672-23 | MARB UIB 1135 | Norway |  |  |
|  | EBAHY008-16 | EBAI-Hyd08 | Norway |  |  |
|  | EBAHY009-16 | EBAI-Hyd09 | Norway |  |  |
|  | MARBN621-23 | MARB UIB 704 | Norway |  |  |
|  | MARBN981-23 | MARB UIB 969 | Norway |  |  |
|  | MARBN679-23 | MARB UIB 1142 | Norway |  |  |
|  | MARBN598-23 | MARB UIB 681 | Norway |  |  |
|  | MARBN822-23 | MARB UIB 1095 | Norway |  |  |
|  | MARBN1025-23 | MARB UIB 1013 | Norway |  |  |
|  | MARBN1026-23 | MARB UIB 1014 | Norway |  |  |
|  | HYDAL067-23 | CCDB-44301-F07 | Belgium |  |  |
|  | HYDAL068-23 | CCDB-44301-F08 | Belgium |  |  |
| *Sperchon squamosus* | MMHYD180-19 | HYDCA314 | Norway | BOLD:AEB7886 |  |
| *Sperchon turgidus* | NLACA092-15 | RMNH.ACA.1201 | Netherlands | BOLD:ACS0295 |  |
|  | NLACA100-15 | RMNH.ACA.1220 | Netherlands |  |  |
| *Sperchon longirostris* | LBCWS078-19 | CrenoBarcode H224 | Austria | BOLD:ADU1021 |  |
|  | LBCWS079-19 | CrenoBarcode H225 | Austria |  |  |
|  | LBCWS083-19 | CrenoBarcode H230 | Austria |  |  |
|  | *LBCWS084-19* | CrenoBarcode H231 | Austria |  |  |
|  | *LBCWS085-19* | CrenoBarcode H232 | Austria |  |  |
| *Sperchon mutilus* | LBCWS064-19 | CrenoBarcode H189 | Italy | BOLD:ADS9620 |  |
|  | LBCWS065-19 | CrenoBarcode H190 | Italy |  |  |
| *Sperchon kyrgyzicus* | NLACA1113-17 | RMNH.509176 | Kyrgyzstan | BOLD:ADF5325 |  |
| *Sperchon thienemanni* | LBCWS158-19 | CrenoBarcode H386 | Austria | BOLD:ADV4077 |  |
|  | LBCWS159-19 | CrenoBarcode H387 | Austria |  |  |
|  | LBCWS157-19 | CrenoBarcode H384 | Austria |  |  |
|  | LBCWS156-19 | CrenoBarcode H383 | Switzerland |  |  |
|  | LBCWS034-19 | CrenoBarcode H110 | Switzerland |  |  |
|  | HYDAS071-22 | CCDB 39397 F11 | Austria |  |  |
|  | HYDAS141-22 | CCDB 39398 D10 | Austria |  |  |
|  | DNAEC078-20 | Hyd MN VP4 | Montenegro |  |  |
|  | DNAEC083-20 | Hyd MN VP10 | Montenegro |  |  |
|  | HYDOC034-22 | CCDB 44300 C10 | Montenegro |  |  |
|  | NOVMB094-21 | CCDB 38559 H10 | France | BOLD:AEO5165 |  |
|  | NOVMB095-21 | CCDB 38559 H11 | France |  |  |
|  | HYDAS124-22 | CCDB 39398 C05 | Austria | BOLD:AER8061 |  |
|  | HYDAS150-22 | CCDB 39398 E07 | Austria |  |  |
|  | HYDAS113-22 | CCDB 39398 B06 | Austria |  |  |
|  | HYDAS095-22 | CCDB 39397 H11 | Austria |  |  |
|  | HYDAS093-22 | CCDB 39397 H09 | Austria |  |  |
|  | NLACA427-15 | RMNH.ACA.852 | Netherlands | BOLD:ACR9585 |  |
|  | NLACA428-15 | RMNH.ACA.853 | Netherlands |  |  |
|  | NLACA421-15 | RMNH.ACA.846 | Netherlands | BOLD:ACS0087 |  |
|  | NLACA945-17 | RMNH.5070703 | Netherlands |  |  |
|  | NLACA1054-17 | RMNH.5070812 | Netherlands |  |  |
|  | NLACA055-15 | RMNH.ACA.1073 | Netherlands |  |  |
|  | NLACA426-15 | RMNH.ACA.851 | Netherlands |  |  |
|  | NLACA422-15 | RMNH.ACA.847 | Netherlands |  |  |
|  | NLACA054-15 | MNH.ACA.1072 | Netherlands |  |  |
|  | DCCDB083-21 | CCDB38233 G11 | Bosnia and Herzegovina | BOLD:AEI8945 |  |
|  | HYDAL060-23 | CDB-44301-E12 | Bosnia and Herzegovina |  |  |
|  | HYDCG069-24 | CCDB-48498-F09 | Türkiye | BOLD:AGG3777 |  |
|  | HYDCG003-24 | CCDB-48498-A03 | Türkiye | BOLD:AES4247 |  |
|  | HYDCG007-24 | CCDB-48498-A07 | Türkiye |  |  |
|  | HYDAS111-22 | CCDB 39398 B04 | Türkiye |  |  |
|  | HYDAS117-22 | CCDB 39398 B10 | Austria |  |  |
|  | HYDAS111-22 | CCDB 39398 B04 | Austria |  |  |
|  | HYDAS114-22 | CCDB 39398 B07 | Austria |  |  |
|  | HYDAS131-22 | CCDB 39398 C12 | Austria |  |  |
| *Sperchon grigorievka* | NLACA1099-17 | RMNH.5091751 | Kyrgyzstan | BOLD:ADF8466 |  |
| *Sperchon vaginosus* | NLACA417-15 | RMNH.ACA.841 | Netherlands | BOLD:ACS0203 |  |
|  | NLACA944-17 | RMNH.5070702 | Netherlands |  |  |

List of *Atractides* specimens used for building the Neighbour-Joining (NJ) tree (Fig. 5).

| *Atractides nodipalpis* | MARBN1081-23 | MARB UIB 594 | Norway | BOLD:ACR0209 |
| --- | --- | --- | --- | --- |
|  | MARBN1310-23 | MARB UIB 918 | Norway |  |
|  | MARBN1183-23 | MARB UIB 791 | Norway |  |
|  | MARBN836-23 | MARB UIB 1109 | Norway |  |
|  | MARBN835-23 | MARB UIB 1108 | Norway |  |
|  | HYDCA069-15 | HYDCA69 | Norway |  |
|  | MARBN838-23 | MARB UIB 1111 | Norway |  |
|  | MARBN1147-23 | MARB UIB 755 | Norway |  |
|  | MARBN1184-23 | MARB UIB 792 | Norway |  |
|  | MMHYD080-19 | HYDCA214 | Norway |  |
|  | MMHYD081-19 | HYDCA215 | Norway |  |
|  | MARBN604-23 | MARB UIB 687 | Norway |  |
|  | MARBN1171-23 | MARB UIB 779 | Norway |  |
|  | MARBN1291-23 | MARB UIB 899 | Norway |  |
|  | MARBN1309-23 | MARB UIB 917 | Norway |  |
|  | MMHYD038-19 | HYDCA172 | Norway |  |
|  | MARBN1165-23 | MARB UIB 773 | Norway |  |
|  | MARBN1232-23 | MARB UIB 840 | Norway |  |
|  | MARBN1164-23 | MARB UIB 772 | Norway |  |
|  | MARBN873-23 | MARB UIB 1241 | Norway |  |
|  | MARBN874-23 | MARB UIB 1242 | Norway |  |
|  | MARBN974-23 | MARB UIB 962 | Norway |  |
|  | MARBN975-23 | MARB UIB 963 | Norway |  |
|  | MARBN837-23 | MARB UIB 1110 | Norway |  |
|  | MARBN603-23 | MARB UIB 686 | Norway |  |
|  | MARBN1161-23 | MARB UIB 769 | Norway |  |
|  | MARBN1287-23 | MARB UIB 895 | Norway |  |
|  | MMHYD066-19 | HYDCA200 | Norway |  |
|  | MMHYD162-19 | HYDCA296 | Norway |  |
|  | MMHYD443-21 | HYDCA577 | Norway |  |
|  | MMHYD444-21 | \|HYDCA578 | Germany |  |
|  | NLACA452-15 | RMNH.ACA.878 | Netherlands |  |
|  | NLACA943-17 | RMNH.5070701 | Netherlands |  |
|  | NLACA993-17 | RMNH.5070751 | Netherlands |  |
|  | NLACA1121-17 | RMNH.ACA.1536 | Netherlands |  |
|  | NLACA1118-17 | RMNH.ACA.1533 | Netherlands |  |
|  | NLACA991-17 | \|RMNH.5070749 | Netherlands |  |
|  | NLACA408-15 | RMNH.ACA.829\| | Netherlands |  |
|  | NLACA992-17 | RMNH.5070750 | Netherlands |  |
|  | NLACA1119-17 | RMNH.ACA.1534 | Netherlands |  |
|  | NLACA1120-17 | RMNH.ACA.1535 | Netherlands |  |
|  | NLACA1084-17 | RMNH.5091736\| | Netherlands |  |
|  | NLACA1053-17 | RMNH.5070811 | Netherlands |  |
|  | HYDAL076-23 | CCDB-44301-G04 | Belgium |  |
|  | HYDCA065-15 | HYDCA65 | Greenland |  |
|  | DCCDB034-21 | CCDB38233 C10 | Serbia |  |
|  | DNCBD090-20 | CCDB-3867-H06 | Serbia |  |
|  | HYDOC024-22 | CCDB 44300 B12 | Serbia |  |
|  | DCCDB033-21 | CCDB38233 C09 | Serbia |  |
|  | DNCBD067-20 | CCDB-3867-F07 | Montenegro |  |
|  | HYDCG163-24 | CCDB-48501-F08 | Bosnia and Herzegovina |  |
|  | DNAEC072-20 | 41. M19 29A 1 G5 | Montenegro | BOLD:AGH6573 |
|  | HYDCG159-24 | CCDB-48501-F04 | Bosnia and Herzegovina |  |
|  | HYDCG160-24 | CCDB-48501-F05 | Bosnia and Herzegovina |  |
|  | MARBN704-23 | MARB UIB 1167 | Norway | BOLD:AFD5286 |
|  | BSNTN856-23 | BGE 00228 A01 | Portugal | BOLD:AFV2009 |
| *Atractides cf. nodipalpis* | MARBN1153-23 | MARB UIB 761 | Norway | BOLD:AFA3227 |
|  | MARBN1162-23 | MARB UIB 770 | Norway |  |
|  | MARBN1169-23 | MARB UIB 777 | Norway |  |
| *Atractides robustus* | DCDDJ059-21 | CCDB 38361 E11 | Türkiye | BOLD:AEK3669 |
|  | HYDCG074-24 | CCDB-48498-G02 | Türkiye |  |
|  | HYDCG040-24 | CCDB-48498-D04 | Türkiye | BOLD:AGH5609 |
|  | DCCDB065-21 | CCDB38233 F05 | Iran | BOLD:AEI1810 |
|  | IRANM012-20 | 19. IR17 2017 H10 | Iran | BOLD:AED3548 |
| *Atractides robustus* | BSNTN1029-23 | BGE 00227 G07 | Portugal | BOLD:AFF2463 |
|  | DNCBD048-20 | CCDB-3867-D12 | Montenegro | BOLD:ADZ9348 |
|  | DNCBD047-20 | CCDB-3867-D11 | Montenegro |  |
|  | DCDDJ086-21 | CCDB 38361 H02 | Montenegro |  |
|  | DCCDB013-21 | CCDB38233 B01 | Montenegro |  |
|  | DNCBD046-20 | CCDB-3867-D10 | Montenegro |  |
|  | HYDCG146-24 | CCDB-48501-E03 | Bosnia and Herzegovina |  |
|  | HYDCG147-24 | CCDB-48501-E04 | Bosnia and Herzegovina |  |
|  | HYDBH010-22 | CCDB 41824 A10\|\| | Bosnia and Herzegovina |  |
|  | HYDAS073-22 | CCDB 39397 G01 | Austria |  |
|  | HYDAS116-22 | CCDB 39398 B09 | Austria |  |
| *Atractides disabatinoi* | HYDAL091-23 | CCDB-44301-H07 | Italy | BOLD:AFC4126 |
|  | HYDAL093-23 | CCDB-44301-H09 | Italy |  |
|  | HYDAL090-23 | CCDB-44301-H06 | Italy |  |
| *Atractides ruffoi* | NOVMB009-21 | CCDB 38559 A09 | France | BOLD:AEN9154 |
| *Atractides marizae* | HYDAS028-22 | CCDB 39397 C04 | Portugal | BOLD:AER7878 |
|  | BSNTN1021-23 | BGE 00227 F11 | Portugal |  |
|  | HYDAS026-22 | CCDB 39397 C02 | Portugal |  |
|  | BSNTN903-23 | BGE 00228 D12 | Portugal |  |
|  | BSNTN914-23 | BGE 00228 E11 | Portugal |  |
|  | BSNTN863-23 | BGE 00228 A08 | Portugal |  |
|  | HYDAS024-22 | CCDB 39397 B12 | Portugal |  |
|  | HYDAS029-22 | CCDB 39397 C05 | Portugal |  |
|  | BSNTN922-23 | BGE 00228 F07 | Portugal |  |
| *Atractides milosevici* | MARBN1307-23 | MARB UIB 915 | Norway | BOLD:AED3547 |
|  | DNAEC071-20 | 40. M19 29A 1 G4 | Montenegro |  |
|  | DNCBD065-20 | CCDB-3867-F05 | Montenegro |  |
| *Atractides subasper* | HYDOC007-22 | CCDB 44300 A07 | Serbia | BOLD:AEX4044 |
|  | HYDCG086-24 | CCDB-48498-H02 | Türkiye | BOLD:AGG3778 |
| *Atractides subasper (Sardinia)* | HYDBH064-22 | CCDB 41824 F04 | Italy | BOLD:AES6460 |
|  | HYDAL089-23 | CCDB-44301-H05 | Italy |  |
|  | HYDAL094-23 | CCDB-44301-H10 | Italy |  |
| *Atractides gibberipalpis* | HYDBH003-22 | CCDB 41824 A03 | Montenegro | BOLD:AFI2792 |
|  | HYDBH004-22 | CCDB 41824 A04 | Montenegro |  |
|  | DCCDB014-21 | CCDB38233 B02 | Montenegro |  |
|  | DCDDJ031-21 | CCDB 38361 C07 | Montenegro | BOLD:AEK7766 |
|  | HYDAS122-22 | CCDB 39398 C03 | Austria | BOLD:AES8583 |
|  | LBCWS219-19 | CrenoBarcode H508 | Germany | BOLD:ADU2475 |
|  | HYDBH001-22 | CCDB 41824 A01 | Montenegro |  |
|  | NOVMB070-21 | CCDB 38559 F10 | France | BOLD:AEO4226 |
|  | NOVMB032-21 | CCDB 38559 C08 | France |  |
|  | NOVMB082-21 | CCDB 38559 G10 | France |  |
|  | NOVMB044-21 | CCDB 38559 D08 | France |  |
|  | NOVMB071-21 | CCDB 38559 F11 | France |  |
|  | NOVMB010-21 | CCDB 38559 A10 | France |  |
|  | NOVMB091-21 | CCDB 38559 H07 | France |  |
|  | NOVMB019-21 | CCDB 38559 B07 | France |  |
|  | NOVMB081-21 | CCDB 38559 G09 | France |  |
| *Atractides tener* | MARBN654-23 | MARB UIB 737 | Norway | BOLD:ACG4776 |
|  | MARBN1005-23 | MARB UIB 993 | Norway |  |
|  | MARBN869-23 | MARB UIB 1237 | Norway |  |
|  | MARBN1080-23 | MARB UIB 593 | Norway |  |
|  | MARBN1308-23 | MARB UIB 916 | Norway |  |
|  | MARBN1285-23 | MARB UIB 893 | Norway |  |
|  | MARBN1198-23 | MARB UIB 806 | Norway |  |
|  | MARBN1114-23 | MARB UIB 627 | Norway |  |
|  | MARBN1052-23 | MARB UIB 565 | Norway |  |
|  | MARBN1000-23 | MARB UIB 988 | Norway |  |
|  | MARBN969-23 | MARB UIB 957 | Norway |  |
|  | MARBN968-23 | MARB UIB 956\| | Norway |  |
|  | MARBN806-23 | MARB UIB 1079 | Norway |  |
|  | MARBN734-23 | MARB UIB 1197 | Norway |  |
|  | MARBN578-23 | MARB UIB 661 | Norway |  |
|  | MARBN1186-23 | MARB UIB 794 | Norway |  |
|  | MARBN1078-23 | MARB UIB 591 | Norway |  |
|  | MARBN919-23 | MARB UIB 1287 | Norway |  |
|  | MARBN1227-23 | MARB UIB 835 | Norway |  |
|  | MARBN698-23 | MARB UIB 1161 | Norway |  |
|  | MARBN805-23 | MARB UIB 1078 | Norway |  |
|  | MARBN817-23 | MARB UIB 1090 | Norway |  |
|  | MARBN848-23 | MARB UIB 1121 | Norway |  |
|  | MARBN868-23 | MARB UIB 1236 | Norway |  |
|  | MARBN1129-23 | MARB UIB 642 | Norway |  |
|  | MARBN608-23 | MARB UIB 691 | Norway |  |
|  | MARBN1079-23 | MARB UIB 592 | Norway |  |
|  | MARBN1197-23 | MARB UIB 805 | Norway |  |
|  | MARBN653-23 | MARB UIB 736 | Norway |  |
|  | MARBN1115-23 | MARB UIB 628 | Norway |  |
|  | MARBN1072-23 | MARB UIB 585 | Norway |  |
|  | HYDCA104-18 | HYDCA104 | Norway |  |
|  | MARBN735-23 | MARB UIB 1198 | Norway |  |
|  | MARBN999-23 | MARB UIB 987 | Norway |  |
|  | MARBN699-23 | MARB UIB 1162 | Norway |  |
| *Atractides nodipalpis* | MMHYD129-19 | HYDCA263 | Norway | BOLD:ADZ1306 |
|  | MARBN978-23 | MARB UIB 966 | Norway |  |
|  | MARBN803-23 | MARB UIB 1076 | Norway |  |
|  | MARBN683-23 | MARB UIB 1146 | Norway |  |
|  | MARBN682-23 | MARB UIB 1145 | Norway |  |
|  | MMHYD085-19 | HYDCA219\| | Norway |  |
|  | MARBN1049-23 | MARB UIB 562 | Norway |  |
|  | MARBN1166-23 | MARB UIB 774 | Norway |  |
|  | MARBN1223-23 | MARB UIB 831 | Norway |  |
|  | MARBN1224-23 | MARB UIB 832 | Norway |  |
|  | MARBN1226-23 | MARB UIB 834 | Norway |  |
|  | MARBN1319-23 | MARB UIB 927 | Norway |  |
|  | MARBN697-23 | MARB UIB 1160 | Norway |  |
|  | MARBN1048-23 | MARB UIB 561 | Norway | BOLD:ADY7881 |
|  | MARBN1284-23 | MARB UIB 892 | Norway |  |
|  | MARBN1167-23 | MARB UIB 775 | Norway |  |
|  | MARBN898-23 | MARB UIB 1266 | Norway |  |
|  | MMHYD086-19 | HYDCA220 | Norway |  |
|  | MMHYD217-20 | HYDCA353 | Norway |  |
|  | MARBN804-23 | MARB UIB 1077 | Norway |  |
|  | MARBN607-23 | MARB UIB 690 | Norway |  |
|  | MARBN920-23 | MARB UIB 1288 | Norway |  |
|  | MARBN696-23 | MARB UIB 1159 | Norway |  |
|  | MARBN801-23 | MARB UIB 1074 | Norway |  |
|  | MARBN1168-23 | MARB UIB 776 | Norway |  |
|  | MMHYD219-20 | HYDCA355 | Norway | BOLD:ADP2485 |
|  | MARBN572-23 | MARB UIB 655 | Norway |  |
|  | MARBN802-23 | MARB UIB 1075 | Norway |  |
|  | MARBN1320-23 | MARB UIB 928 | Norway |  |
|  | MMHYD197-20 | HYDCA331 | Norway |  |
|  | MARBN816-23 | MARB UIB 1089 | Norway |  |
|  | MMHYD084-19 | HYDCA218 | Norway |  |
|  | MMHYD140-19 | \|HYDCA274 | Norway |  |
|  | MMHYD153-19 | HYDCA287 | Norway |  |
|  | MARBN938-23 | MARB UIB 1306 | Norway |  |
|  | MARBN1196-23 | MARB UIB 804 | Norway |  |
|  | MARBN1163-23 | MARB UIB 771 | Norway |  |
|  | MMHYD115-19 | HYDCA249\| | Norway |  |
|  | MMHYD137-19 | HYDCA271 | Norway |  |
|  | HYDCA130-18 | \|HYDCA130 | Norway |  |
|  | MMHYD138-19 | HYDCA272 | Norway |  |
|  | MMHYD139-19 | HYDCA273 | Norway |  |
|  | MMHYD220-20 | HYDCA356 | Norway |  |
|  | MMHYD338-21 | HYDCA472\| | Norway |  |
|  | MARBN571-23 | MARB UIB 654 | Norway |  |
|  | MARBN778-23 | MARB UIB 1051 | Norway |  |
|  | MARBN944-23 | MARB UIB 1312 | Norway |  |
|  | MARBN1073-23 | MARB UIB 586 | Norway |  |
|  | MARBN1170-23 | MARB UIB 778 | Norway |  |
|  | MARBN1251-23 | MARB UIB 859 | Norway |  |
|  | MARBN1286-23 | MARB UIB 894 | Norway |  |
|  | MARBN1292-23 | MARB UIB 900 | Norway |  |
|  | LBCWS237-19 | CrenoBarcode H534 | Germany |  |
|  | LBCWS238-19 | CrenoBarcode H535 | Germany |  |
| *Atractides protendens* | LBCWS137-19 | CrenoBarcode H336 | Germany | BOLD:ADT4472 |
|  | LBCWS138-19 | CrenoBarcode H337 | Germany |  |
| *Atractides inflatus* | HYDIR026-23 | CCDB 39399 C02 | Türkiye | BOLD:ACB4677 |
|  | HYDIR033-23 | CCDB 39399 C09 | Türkiye |  |
|  | SEPTB021-21 | CCDB 38362 B09 | Greece |  |
|  | HYDIR032-23 | CCDB 39399 C08 | Türkiye |  |
|  | DCDDJ061-21 | CCDB 38361 F01 | Türkiye |  |
|  | HYDIR031-23 | CCDB 39399 C07 | Türkiye |  |
|  | DNAEC052-20 | 14. M19 12 4 E5 | Montenegro |  |
|  | HYDIR028-23 | CCDB 39399 C04 | Türkiye |  |
|  | SEPTB060-21 | CCDB 38362 E12 | Greece |  |
|  | DCDDJ060-21 | CCDB 38361 E12 | Türkiye |  |
|  | DCDDJ058-21 | CCDB 38361 E10 | Türkiye |  |
|  | NOVMB013-21 | CCDB 38559 B01 | France |  |
|  | HYDIR034-23 | CCDB 39399 C10 | Türkiye |  |
|  | BSNTN898-23 | BGE 00228 D07 | Portugal |  |
|  | BBIOP043-24 | BGE 00109 D07 | Portugal |  |
|  | BSNTN528-23 | BGE 00110 E05 | Portugal | BOLD:AFI9009 |
| *Atractides inflatus (Corsica)* | NOVMB040-21 | CCDB 38559 D04 | France | BOLD:AEO0988 |
| *Atractides nahavandii* | IRANM005-20 | 9. IR8 2017 H3 | Iran | BOLD:AED4001 |
| *Atractides pumilus* | HYDAL035-23 | CCDB-44301-C11 | Bosnia and Herzegovina | BOLD:AFD7424 |
|  | HYDAL052-23 | CCDB-44301-E04 | Bosnia and Herzegovina |  |
| *Atractides pumilus* (Corsica) | NOVMB065-21 | CCDB 38559 F05 | France | BOLD:AEN9335 |
| *Atractides latipalpis* | DCDDJ073-21 | CCDB 38361 G01 | Germany | BOLD:AEK4539 |
| *Atractides samsoni* | MMHYD337-21 | HYDCA471 | Norway | BOLD:AEA3265 |
|  | MARBN1185-23 | MARB UIB 793 | Norway |  |
|  | MMHYD336-21 | HYDCA470 | Norway |  |
|  | MMHYD334-21 | HYDCA468 | Norway |  |
|  | MMHYD335-21 | HYDCA469 | Norway |  |
| *Atractides adnatus* | LBCWS126-19 | CrenoBarcode H292 | Germany | BOLD:ADU1763 |
|  | LBCWS127-19 | CrenoBarcode H293 | Germany |  |
|  | LBCWS125-19 | CrenoBarcode H291 | Germany |  |
| *Atractides separatus* | LBCWS093-19 | CrenoBarcode H243 | Germany | BOLD:ADV7653 |
|  | LBCWS129-19 | CrenoBarcode H311 | Austria |  |
| *Atractides spinipes* | HYDAS082-22 | CCDB 39397 G10 | Austria | BOLD:AET7894 |
|  | HYDAS162-22 | CCDB 39398 F07 | Austria |  |
| *Atractides vaginalis* | LBCWS029-19 | CrenoBarcode H099 | Switzerland | BOLD:ADU6015 |
|  | LBCWS033-19 | CrenoBarcode H108 | Switzerland |  |
|  | LBCWS025-19 | CrenoBarcode H078 | Switzerland |  |
|  | LBCWS021-19 | CrenoBarcode H073 | Switzerland |  |
|  | LBCWS030-19 | CrenoBarcode H100 | Switzerland |  |
|  | LBCWS022-19 | CrenoBarcode H074 | Switzerland |  |
|  | BCWS094-19 | CrenoBarcode H244 | Germany |  |
|  | LBCWS063-19 | CrenoBarcode H186 | Italy |  |
| *Atractides walteri* | LBCWS173-19 | CrenoBarcode H445 | Austria | BOLD:ADV7370 |
|  | LBCWS183-19 | CrenoBarcode H459 | Austria |  |
|  | LBCWS175-19 | CrenoBarcode H447 | Austria |  |
|  | LBCWS176-19 | CrenoBarcode H448 | Austria |  |
|  | LBCWS128-19 | CrenoBarcode H304 | Austria |  |
|  | LBCWS177-19 | CrenoBarcode H449 | Austria |  |
|  | LBCWS174-19 | CrenoBarcode H446 | Austria |  |
| *Atractides brendle* | LBCWS236-19 | CrenoBarcode H533 | Germany | BOLD:ADT1697 |
|  | LBCWS244-19 | CrenoBarcode H548 | Germany |  |
|  | LBCWS235-19 | CrenoBarcode H532 | Germany |  |
|  | LBCWS243-19 | CrenoBarcode H547 | Germany |  |
| *A.macrolaminatus/*  *loricatus* | LBCWS178-19 | CrenoBarcode H450 | Austria | BOLD:ADU8387 |
| *Atractides blongus* | DCDDJ081-21 | CCDB 38361 G09 | Austria | BOLD:AEL4741 |
| *A. macrolaminatus* | DCDDJ075-21 | CCDB 38361 G03 | Austria | BOLD:AEK7765 |
| *Atractides sp. B* sensu Blattner et al. 2019 | LBCWS234-19 | CrenoBarcode H531 | Germany | \|BOLD:ADU6120 |
| *Atractides sp. A* sensu Blattner et al. 2019 | HYDAS178-22 | CCDB 39398 G11 | Austria | BOLD:ADT8145 |
|  | HYDAS177-22 | CCDB 39398 G10 | Austria |  |
|  | LBCWS233-19 | CrenoBarcode H528 | Germany |  |
| *Atractides graecus* | HYDCG067-24 | CCDB-48498-F07 | Türkiye | BOLD:AGG3781 |
| *Atractides stankovici* | DNAEC020-20 | 13. CG2020 4 B10 | Montenegro | BOLD:AED3550 |
|  | DNAEC021-20 | 14. CG2020 4 B11 | Montenegro |  |
|  | DCCDB032-21 | CCDB38233 C08 | Montenegro |  |
|  | DCCDB031-21 | CCDB38233 C07 | Montenegro |  |
|  | DNCBD095-20 | CCDB-3867-H11 | North Macedonia |  |
| *Atractides mossahebii* | IRANM013-20 | 20. IR17 2017 H11 | Iran | BOLD:AED7021 |
|  | RANM014-20 | 21. IR7 2017 H12 | Iran |  |
| *Atractides hyrcaniensis* | IRANM006-20 | 10. IR2 2017 H4 | Iran | BOLD:AED7022 |
|  | RANM007-20 | 11. IR2 2017 H5 | Iran |  |
| *Atractides cultellatus* | HYDAS023-22 | CCDB 39397 B11 | Portugal\| | BOLD:AEU1503 |
| *Atractides fluviatilis* | DNCBD082-20 | CCDB-3867-G10 | Montenegro | BOLD:AEF1143 |
|  | HYDOC075-22 | CCDB 44300 G03 | Croatia |  |
|  | HYDOC076-22 | CCDB 44300 G04 | Croatia |  |
|  | HYDOC080-22\| | CCDB 44300 G08\|\| | Croatia |  |
| *Atractides inflatipalpis* | HYDCG026-24 | CCDB-48498-C02 | Türkiye | BOLD:AGG3787 |
|  | HYDCG024-24 | CCDB-48498-B12 | Türkiye |  |
|  | DNCBD020-20 | CCDB-3867-B08 | Montenegro | BOLD:AEF1145 |
| *Atractides zagrosensis* | HYDIR038-23 | CCDB 39399 D02 | Iran | BOLD:AFF9958 |
|  | HYDIR048-23 | CCDB 39399 D12 | Iran |  |
|  | HYDIR046-23 | CCDB 39399 D10 | Iran |  |
|  | HYDIR027-23 | CCDB 39399 C03 | Iran |  |
| *Atractides corsicus* | HYDBH039-22 | CCDB 41824 D03 | Italy | BOLD:AET7893 |
|  | NOVMB015-21 | CCDB 38559 B03 | France | BOLD:AEO5312 |
|  | NOVMB087-21 | CCDB 38559 H03 | France |  |
| *Atractides lunipes* | HYDCG068-24 | CCDB-48498-F08 | Türkiye | BOLD:AGG3780 |
| *Atractides ovalis* | MMHYD202-20 | HYDCA336 | Norway | BOLD:AEC4631 |
| *Atractides distans* | NLACA438-15 | \|RMNH.ACA.864 | Netherlands | BOLD:ACS0163 |
|  | NLACA439-15 | RMNH.ACA.865 | Netherlands |  |
|  | NLACA414-15 | RMNH.ACA.838 | Netherlands |  |
|  | NLACA413-15 | RMNH.ACA.837 | Netherlands |  |
|  | NLACA412-15 | RMNH.ACA.836 | Netherlands |  |
| *Atractides nikooae* | HYDCG058-24 | CCDB-48498-E10 | Turkey | BOLD:AGG3766 |
|  | HYDCG046-24 | CCDB-48498-D10 | Turkey |  |
|  | HYDCG047-24 | CCDB-48498-D11 | Turkey |  |
| *Atractides allgaier* | HYDAS009-22 | CCDB 39397 A09 | Portugal | BOLD:AEU1287 |
|  | HYDAS014-22 | CCDB 39397 B02 | Portugal |  |
| *Atractides panniculatus* | LBCWS141-19 | CrenoBarcode H364 | Germany | BOLD:ADU8027 |
|  | LBCWS145-19 | CrenoBarcode H368 | Germany |  |
|  | LBCWS144-19 | CrenoBarcode H367 | Germany |  |
|  | LBCWS143-19 | CrenoBarcode H366 | Germany |  |
|  | LBCWS142-19 | CrenoBarcode H365 | Germany |  |
| *Atractides rivalis* | HYDME026-22 | CCDB 41823 C02 | Germany | BOLD:ADG8744 |
|  | HYDME017-22 | CCDB 41823 B05 | Germany |  |
|  | HYDME018-22 | CCDB 41823 B06 | Germany |  |
| *Atractides fissus* | LBCWS198-19 | CrenoBarcode H483 | Austria | BOLD:ADU0063 |
|  | LBCWS199-19 | CrenoBarcode H484 | Austria |  |
|  | DCCDB015-21 | CCDB38233 B03 | Montenegro | BOLD:AEI1811 |
|  | DCCDB046-21 | CCDB38233 D10 | Montenegro |  |
|  | HYDBH005-22\| | CCDB 41824 A05 | Montenegro |  |
| *Atractides fonticolus* | LBCWS202-19 | CrenoBarcode H488 | Germany | BOLD:ADS3489 |
|  | LBCWS203-19 | CrenoBarcode H489 | Germany |  |
|  | LBCWS201-19 | CrenoBarcode H487 | Germany |  |
|  | DCCDB020-21 | CCDB38233 B08 | Montenegro | BOLD:AGH7558 |
|  | DCCDB021-21 | CCDB38233 B09 | Montenegro |  |
|  | HYDCG023-24 | CCDB-48498-B11 | Türkiye | BOLD:AGG3788 |
|  | HYDCG103-24 | CCDB-48501-A08 | Türkiye |  |
| *Atractides remotus* | HYDAL075-23 | CCDB-44301-G03 | Belgium | BOLD:AFE0132 |
|  | HYDAL077-23 | CCDB-44301-G05 | Belgium | BOLD:AFC6398 |
|  | BBIOP090-24 | BGE 00109 H06 | Portugal | BOLD:AFW2656 |
| *Atractides anae* | DNAEC014-20 | 1. CG2020 8 B3 | Montenegro | BOLD:AED1201 |
| *Atractides pennatus* | HYDME006-22 | CCDB 41823 A06 | Germany | BOLD:AES4768 |
|  | HYDCG151-24 | CCDB-48501-E08 | Bosnia and Herzegovina\| |  |
|  | HYDCG127-24 | CCDB-48501-C08\| | Bosnia and Herzegovina\| |  |
|  | LBCWS220-19 | CrenoBarcode H509 | Germany | BOLD:ADV9389 |
|  | HYDAS133-22 | CCDB 39398 D02 | Austria |  |
|  | HYDCG125-24 | CCDB-48501-C06 | Bosnia and Herzegovina | BOLD:AGH5610 |
|  | HYDCG139-24 | CCDB-48501-D08 | Bosnia and Herzegovina |  |
|  | HYDCG126-24 | CCDB-48501-C0 | Bosnia and Herzegovina | BOLD:AGH5611 |
|  | SEPTA024-21 | CCDB 38363 B12 | Montenegro | BOLD:ADF7007 |
|  | SEPTA025-21 | CCDB 38363 C01 | Montenegro |  |
|  | DNAEC015-20 | 3. CG2020 2 B4\|\| | Montenegro |  |
|  | DNAEC027-20 | 23. CG2020 9 C5\|\| | Montenegro |  |
|  | DNAEC028-20 | 25. CG2020 9 C6 | Montenegro |  |
|  | DNAEC042-20 | 4. M19 22 1 D8 | Montenegro |  |
|  | DNAEC066-20 | 31. M19 23 1 F9 | Montenegro |  |
|  | DNAEC067-20 | 32. M19 23 1 F10 | Montenegro |  |
|  | DNCBD009-20 | CCDB-38679-A09 | Montenegro |  |
|  | NLACA1085-17 | RMNH.5091737 | Netherlands |  |
|  | LBCWS221-19 | CrenoBarcode H510 | Germany |  |
| *Atractides latipes* | DNAEC056-20 | 18. M19 08B 7 E9 | Montenegro | BOLD:AED4000 |
| *Atractides inflatipes* | DNCBD075-20 | CCDB-3867-G03 | Montenegro | BOLD:AEF1144 |
| *Atractides castor* | HYDBH063-22 | CCDB 41824 F03 | Italy | BOLD:AET6487 |
|  | HYDBH071-22 | CCDB 41824 F11 | Italy |  |
|  | HYDBH072-22 | CCDB 41824 F12 | Italy |  |
| *Atractides acutirostris* | SEPTB082-21 | CCDB 38362 G10 | Croatia | BOLD:AEN8023 |
|  | HYDIR025-23 | CCDB 39399 C01 | Türkiye | BOLD:AFG8047 |
|  | HYDIR029-23 | CCDB 39399 C05 | Türkiye |  |
